# Rapid and sustained homeostatic control of presynaptic exocytosis at a central synapse

**DOI:** 10.1073/pnas.1909675116

**Published:** 2019-11-04

**Authors:** Igor Delvendahl, Katarzyna Kita, Martin Müller

**Affiliations:** ^a^Institute of Molecular Life Sciences, University of Zurich, 8057 Zurich, Switzerland;; ^b^Neuroscience Center Zurich, University of Zurich, ETH Zurich, 8057 Zurich, Switzerland

**Keywords:** homeostatic plasticity, synaptic transmission, exocytosis, mossy fiber, cerebellum

## Abstract

Animal behavior is remarkably robust despite constant changes in neural activity. Homeostatic plasticity stabilizes central nervous system (CNS) function on time scales of hours to days. If and how CNS function is stabilized on more rapid time scales remains unknown. Here, we discovered that mossy fiber synapses in the mouse cerebellum homeostatically control synaptic efficacy within minutes after pharmacological glutamate receptor impairment. This rapid form of homeostatic plasticity is expressed presynaptically. We show that modulations of readily releasable vesicle pool size and release probability normalize synaptic strength in a hierarchical fashion upon acute pharmacological and prolonged genetic receptor perturbation. Presynaptic membrane capacitance measurements directly demonstrate regulation of vesicle pool size upon receptor impairment. Moreover, presynaptic voltage-clamp analysis revealed increased Ca^2+^-current density under specific experimental conditions. Thus, homeostatic modulation of presynaptic exocytosis through specific mechanisms stabilizes synaptic transmission in a CNS circuit on time scales ranging from minutes to months. Rapid presynaptic homeostatic plasticity may ensure stable neural circuit function in light of rapid activity-dependent plasticity.

## Significance

Homeostatic mechanisms stabilize neural activity, and there are genetic links between homeostatic plasticity and neural disease. While homeostatic plasticity in the central nervous system (CNS) operates on relatively slow time scales of hours to days, activity-dependent forms of synaptic plasticity alter neural activity on much faster time scales. It is unclear if homeostatic plasticity stabilizes CNS synapses on rapid time scales. Here, we uncovered that cerebellar synapses stabilize transmission within minutes upon activity perturbation. This is achieved through homeostatic control of presynaptic exocytosis. We show that synergistic modulation of distinct presynaptic mechanisms not only maintains synaptic efficacy on rapid, but also on prolonged time scales. Homeostatic control of presynaptic exocytosis may be a general mechanism for stabilizing CNS function.

Adaptive animal behavior presupposes plastic, and yet stable, neural function. While neural activity is constantly changing because of activity-dependent plasticity (1, 2), robust animal behavior can be maintained for a lifetime. However, stable nervous system function and behavior are not a given, as apparent from pathological states such as epilepsy or migraine (3). A number of studies revealed that homeostatic plasticity actively stabilizes neural excitability and synaptic transmission (4–7). The most widely studied form of homeostatic synaptic plasticity is synaptic scaling—the bidirectional, compensatory regulation of neurotransmitter receptor abundance (5). This postsynaptic type of homeostatic plasticity is typically observed after chronic pharmacological blockade of action potentials (APs) or neurotransmitter receptors in neuronal cell-culture systems (8–10), but also stabilizes AP firing rates after sensory deprivation in vivo (11, 12).

There is also evidence for presynaptic forms of homeostatic synaptic plasticity. Presynaptic homeostatic plasticity (PHP) stabilizes synaptic efficacy at neuromuscular synapses after acute or sustained neurotransmitter receptor perturbation in several species (13–16). In the mammalian central nervous system (CNS), PHP is mostly studied in cell culture after prolonged perturbation of neural activity (7). The expression of PHP depends on cell-culture age, with cultures older than 2 wk compensating for prolonged activity deprivation through concomitant regulation of presynaptic release, postsynaptic receptor abundance, and synapse number (17–20). Despite evidence for compensatory changes in presynaptic structure and function (21–25), it is currently not well understood how these relate to the postsynaptic modifications during synaptic scaling. Moreover, it has remained largely elusive if PHP stabilizes the activity of native neural circuits (7, 26, 27).

The maintenance of robust CNS function is strongly challenged by Hebbian plasticity, which is induced on rapid time scales (2) and eventually destabilizes neuronal activity (28–30). In contrast, the expression of presynaptic and postsynaptic homeostatic plasticity requires perturbation of CNS function for hours to days (7), raising the question if and how CNS function is stabilized on rapid time scales. Given the discrepancy in temporal dynamics, homeostatic plasticity is thought to slowly integrate and normalize fast neural activity changes (6, 10). However, theoretical work implies that the slow time course of homeostatic plasticity is insufficient to prevent instabilities induced by Hebbian plasticity (31).

Here, we define the time course of a fast, presynaptic form of homeostatic plasticity at a cerebellar synapse. Employing presynaptic and postsynaptic whole-cell recordings in acute brain slices, we discover that synergistic modulation of readily releasable vesicle pool size and vesicular release probability through elevated Ca^2+^-current density underlies the homeostatic control of exocytosis from these CNS terminals. Rapid PHP may stabilize synaptic information transfer to balance changes imposed by perturbations or Hebbian plasticity.

## Results

### Rapid, Reversible Homeostatic Release Modulation.

**Fig. 1.**
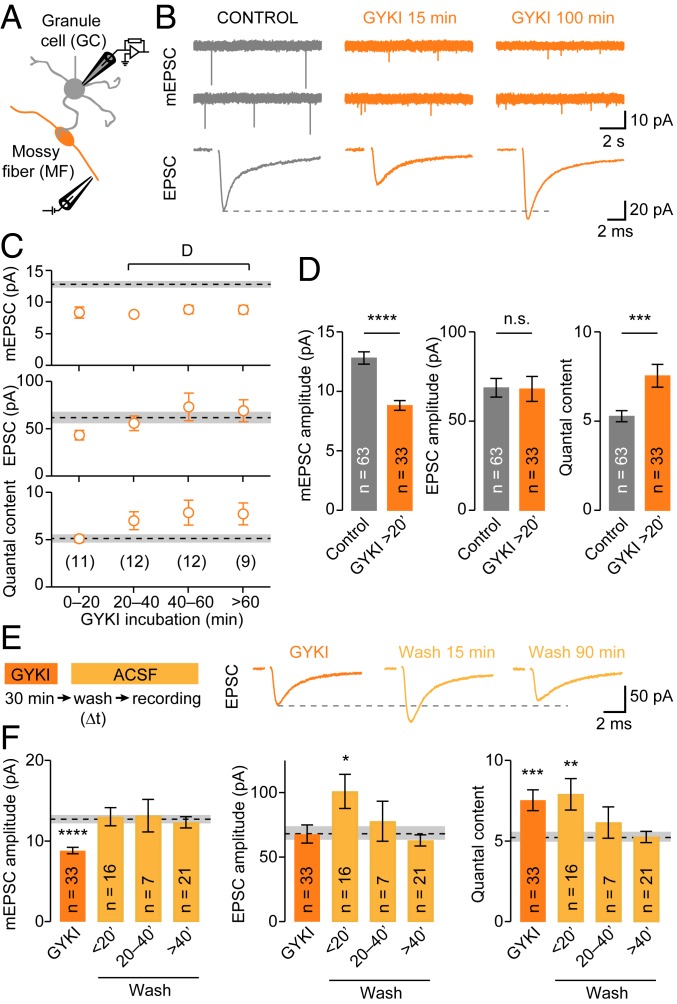
Rapid, reversible homeostatic release modulation in the mouse cerebellum. (*A*) Schematic of recordings from cerebellar GCs and axonal MF stimulation. (*B*) Representative mEPSCs and AP-evoked EPSCs recorded from a control synapse (*Left*), or from synapses incubated with 2 µM of the AMPAR antagonist GYKI 53655 for the indicated incubation times (*Center* and *Right*). (*C*) Average mEPSC amplitude, EPSC amplitude, and quantal content for different GYKI incubation times. Dashed lines and shaded areas represent control values ± SEM, numbers represent individual recordings. (*D*) Average mEPSC amplitude (Cohen’s *d* = −1.1; *P* = 1.7*e*−6), EPSC amplitude (*d* = −0.02; *P* = 0.94), and quantal content (= EPSC/mEPSC; *d* = 0.78; *P* = 0.0005) of all cells with >20 min of GYKI incubation. (*E*, *Left*) Experimental design. Slices were incubated in ACSF containing 2 µM GYKI for 30 min and recordings were subsequently conducted in control ACSF (“wash”) after indicated time intervals (∆*t*). (*E*, *Right*) Example EPSCs for GYKI and washout conditions. (*F*) Average data for mEPSC amplitude, EPSC amplitude, and quantal content. Dashed lines and shaded areas indicate control averages ± SEM.

We probed if fast presynaptic homeostatic mechanisms stabilize synaptic transmission in an acute mouse brain slice preparation. To this end, we recorded miniature excitatory postsynaptic currents (mEPSCs) and AP-evoked excitatory postsynaptic currents (EPSCs) at adult cerebellar mossy fiber (MF)-granule cell (GC) synapses (Fig. 1*A*) (32, 33) after various incubation times with the noncompetitive AMPA receptor (AMPAR) blocker GYKI 53655. GYKI application at subsaturating concentration (2 µM, ref. 34) for 5 to 20 min reduced mEPSC and evoked EPSC amplitudes by ∼30% without affecting their kinetics (Fig. 1 *B* and *C* and *SI Appendix*, Fig. S1 *A*–*G*). By contrast, recordings obtained after 20 min of GYKI incubation revealed EPSC amplitudes that were comparable to control levels (Fig. 1 *B*–*D*). Longer incubation times of up to 100 min gave similar results (Fig. 1 *B*–*D*). Because GYKI equally reduced mEPSC amplitudes at all incubation times, there was a significant increase in quantal content after 20 min (Fig. 1 *C* and *D*), indicative of enhanced neurotransmitter release. The increase in quantal content scaled with the degree of mEPSC reduction, such that EPSC amplitudes were precisely maintained at control levels (*SI Appendix*, Fig. S1*H*), consistent with the phenomenology of PHP at the neuromuscular junction (NMJ) (15, 35). These data demonstrate a rapid form of PHP at mammalian CNS synapses upon acute, subsaturating AMPAR blockade on the minute time scale.

To investigate the reversibility of PHP, we recorded from MF-GC synapses following GYKI washout. Slices were incubated with GYKI for 30 min and then exposed to control artificial cerebrospinal fluid (ACSF) for varying times before obtaining whole-cell recordings (Fig. 1*E*). While mEPSC amplitudes recovered to control levels, EPSC amplitudes, and quantal content remained significantly larger than under control conditions up to ∼20 min after GYKI washout (Fig. 1*F*). This implies that PHP expression persists when AMPAR perturbation is removed on a short time scale. Additional recordings demonstrated that PHP was fully reversible within ∼20–40 min after GYKI washout (Fig. 1*F*), similar to previous reports at the mouse NMJ (16). Thus, PHP completely reverses within tens of minutes after reattained receptor function.

### Acute Homeostatic Readily Releasable Pool Size Modulation.

**Fig. 2.**
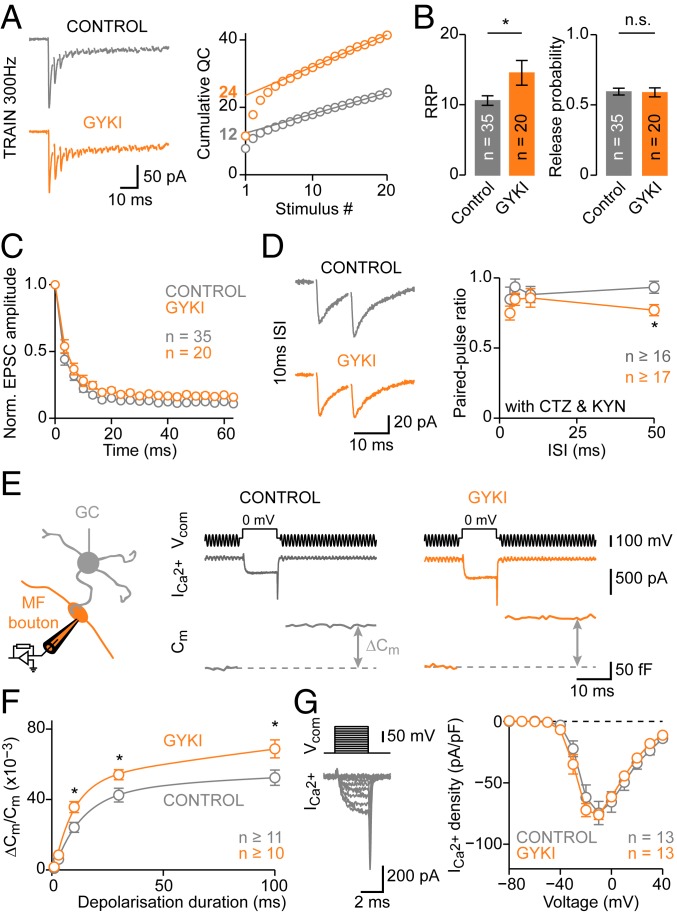
Acute AMPAR perturbation increases RRP size. (*A*, *Left*) Representative recordings of 300-Hz stimulation (20 pulses) for control and GYKI-treated synapses (>20-min incubation). (*A*, *Right*) Cumulative quantal content [calculated as: (cumulative EPSC amplitude)/(mEPSC amplitude)] for the examples at *Left*. Extrapolated linear fits provide the RRP size estimate (indicated). (*B*) Average data for control and GYKI. GYKI enhanced RRP size (*d* = 0.69; *P* = 0.017) without changing release probability [calculated as: (first EPSC amplitude)/(cumulative EPSC amplitude); *d* = −0.04; *P* = 0.89]. (*C*) Average EPSC amplitude during train stimulation, normalized to the first EPSC. (*D*, *Left*) Representative EPSCs evoked by stimulation with 10-ms interstimulus interval (ISI) in the presence of cyclothiazide (CTZ) and kynurenic acid (KYN) to minimize postsynaptic contributions. (*D*, *Right*) Average PPR versus ISI. (*E*, *Left*) Cartoon illustrating presynaptic whole-cell recordings from cerebellar MF boutons. (*E*, *Right*) Voltage command (V_com_, 10-ms depolarization to 0 mV, *Top*), representative pharmacologically isolated Ca^2+^ currents (I_Ca_, *Middle*), and membrane capacitance (C_m_) jumps (*Bottom*) for control and GYKI-treated boutons (>20-min incubation). (*F*) Average C_m_ increase (∆C_m_) versus depolarization duration (η^2^ = 0.14; *P* = 6.6*e*−05; 2-way ANOVA). Lines are biexponential fits; ∆C_m_ is normalized to resting C_m_. (*G*, *Left*) Representative recording of I_Ca_. (*G*, *Right*) I_Ca_ density versus voltage (η^2^ = <0.01; *P* = 0.88; 2-way ANOVA).

To assess the presynaptic mechanisms underlying neurotransmitter release potentiation upon AMPAR perturbation, we tested if changes in the number of readily releasable vesicles (readily releasable pool, RRP) contribute to PHP following GYKI application. To estimate RRP size, we used high-frequency train stimulation and analysis of cumulative EPSC amplitudes (Fig. 2*A*) (36). This analysis gives an effective RRP size estimate assuming that EPSC amplitudes depress because of vesicle pool depletion. Three hundred-hertz stimulation revealed a pronounced increase in RRP size in the presence of GYKI (Fig. 2*B* and *SI Appendix*, Fig. S3 *C*–*E*) that paralleled the time course of PHP induction and reversal (*SI Appendix*, Fig. S3 *A* and *B*). An increase in release probability (*p*_r_) could also contribute to release potentiation during PHP. However, *p*_r_ estimated from EPSC train stimulation was unchanged after GYKI incubation (Fig. 2*B*). Moreover, EPSC amplitude coefficient of variation (CV) and EPSC paired-pulse ratios (PPRs) were unaffected by GYKI (Fig. 2*D* and *SI Appendix*, Fig. S3 *F* and *G*), indicating that *p*_r_ was largely unchanged.

To directly probe presynaptic function during pharmacological AMPAR perturbation, we performed presynaptic whole-cell recordings from MF boutons (Fig. 2*E*) (33) and studied exocytosis employing membrane capacitance (C_m_) measurements. Brief depolarizations under voltage clamp caused Ca^2+^ influx and a jump in C_m_ corresponding to synaptic vesicle exocytosis (Fig. 2*E*), providing a direct estimate of RRP size. MF boutons displayed significantly larger C_m_ jumps upon depolarizations of various durations in the presence of GYKI (>20-min incubation; Fig. 2*F*). The enhanced exocytosis upon GYKI treatment corroborates the increase in RRP size estimated from postsynaptic recordings (Fig. 2*B*). Our presynaptic C_m_ data provide an independent confirmation of changes in RRP size, because postsynaptic RRP size estimates are based on relating evoked EPSCs to mEPSCs, which may involve partially distinct vesicle pools and/or postsynaptic receptors (37, 38) (*SI Appendix*, Fig. S2). While absolute RRP size estimates from presynaptic C_m_ measurements and postsynaptic EPSC analysis differ because of the high bouton-to-granule cell connectivity (33), both approaches revealed a similar relative RRP size increase after GYKI treatment (∼35%; Fig. 2 *B* and *F*).

Next, we investigated pharmacologically isolated presynaptic Ca^2+^ currents (Fig. 2*G*). Ca^2+^-current density, steady-state activation, and activation or deactivation time constants were similar between GYKI-treated and GYKI-untreated MF boutons (Fig. 2*G* and *SI Appendix*, Fig. S3*I*), indicating no major differences in Ca^2+^-channel levels, voltage dependence of Ca^2+^-channel activation, or channel kinetics. These results suggest largely unaltered *p*_r_, in line with postsynaptic recordings (Fig. 2 *B*–*D*). Together, these data establish that acute AMPAR perturbation at a mammalian central synapse leads to enhanced exocytosis and a rapid and reversible expansion of RRP size without apparent *p*_r_ changes.

### Sustained Homeostatic RRP Size Modulation.

**Fig. 3.**
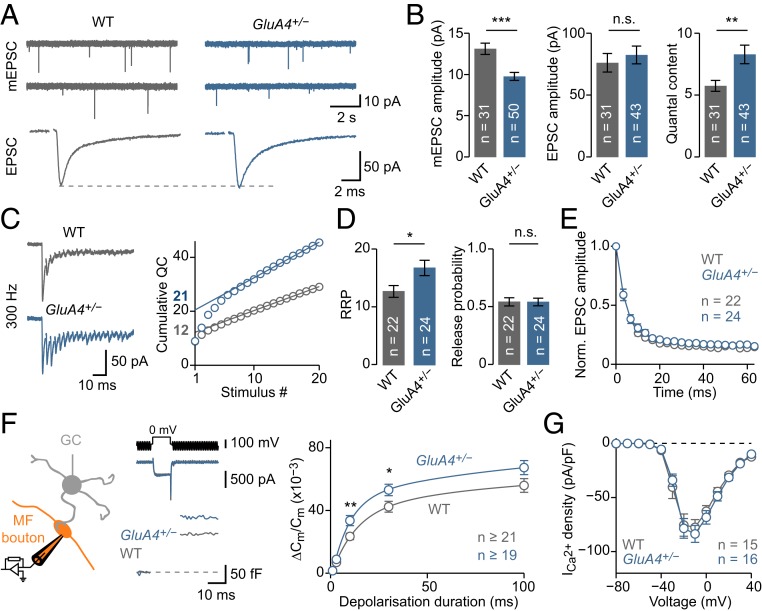
Sustained AMPAR perturbation increases RRP size. (*A*) Representative mEPSCs and AP-evoked EPSCs in WT and *GluA4*^*+/−*^ mice. (*B*) Average mEPSC amplitude (*d* = −0.90; *P* = 0.0001), EPSC amplitude (*d* = 0.14; *P* = 0.55), and quantal content (= EPSC/mEPSC; *d* = 0.70; *P* = 0.0095). (*C*, *Left*) Representative recordings after 300-Hz stimulation. (*C*, *Right*) Cumulative quantal content for the examples at *Left*; estimated RRP size is indicated. (*D*) Average data for WT and *GluA4*^*+/−*^. RRP size was increased at *GluA4*^*+/−*^ synapses (*d* = 0.72; *P* = 0.02) without apparent changes in release probability (*d* = −0.001; *P* = 0.99). (*E*) Average normalized EPSC amplitude during train stimulation. (*F*, *Left*) Schematic of presynaptic recordings. (*F*, *Middle*) Voltage command (V_com_, 10-ms depolarization to 0 mV, *Top*), representative Ca^2+^ currents (I_Ca_, *Middle*), and C_m_ jumps (*F*, *Bottom*) for WT and *GluA4*^*+/−*^. (*F*, *Right*) Average C_m_ increase (∆C_m_) against stimulus duration (η^2^ = 0.07; *P* = 0.0002; 2-way ANOVA). (*G*) I_Ca_ density versus voltage (η^2^ = 0.001; *P* = 0.60; 2-way ANOVA).

To investigate if PHP can be sustained over longer time periods, we recorded from adult (3–10 wk old) heterozygous mice carrying a loss-of-function mutation in *GRIA4*, the gene encoding the GluA4 AMPAR subunit (39, 40). GluA4 confers a large conductance to AMPARs (41) and is expressed in cerebellar GCs (34, 42). Importantly, heterozygous *GRIA4* mice, henceforth called “*GluA4*^*+/−*^”, have reduced GluA4 protein levels (40). *GluA4*^*+/−*^ synapses had significantly smaller mEPSC amplitudes (Fig. 3 *A* and *B* and *SI Appendix*, Figs. S4 *A* and *B* and S5). In contrast, EPSC amplitudes were similar to wild-type (WT) controls (Fig. 3 *A* and *B*), translating into a significant increase in quantal content (Fig. 3*B*). Three hundred-hertz train stimulation revealed a significant increase in effective RRP size at *GluA4*^*+/−*^ synapses (Fig. 3 *C* and *D* and *SI Appendix*, Fig. S4*F*). We did not observe significant changes in *p*_r_ estimated from stimulus trains, PPR, or EPSC amplitude CV between *GluA4*^*+/−*^ and WT synapses (Fig. 3 *D* and *E* and *SI Appendix*, Fig. S4 *D* and *E*). These results provide evidence for increased neurotransmitter release and RRP size after chronic AMPAR impairment.

We then studied exocytosis and presynaptic Ca^2+^ influx after genetic AMPAR perturbation. Presynaptic C_m_ measurements uncovered enhanced exocytosis in *GluA4*^*+/−*^ MF boutons compared to WT (Fig. 3*F*), in agreement with the increase in effective RRP size (Fig. 3*D*). Furthermore, Ca^2+^-current density was similar in both genotypes (Fig. 3*G*), consistent with the *p*_r_ estimates from postsynaptic recordings. These results show that a sustained increase in RRP size—but not *p*_r_—underlies PHP expression upon genetic AMPAR perturbation at MF-GC synapses. As we recorded from adult synapses, our data demonstrate that PHP can be chronically expressed in the mammalian CNS for months.

### Synergistic Homeostatic Control of RRP Size and Release Probability.

**Fig. 4.**
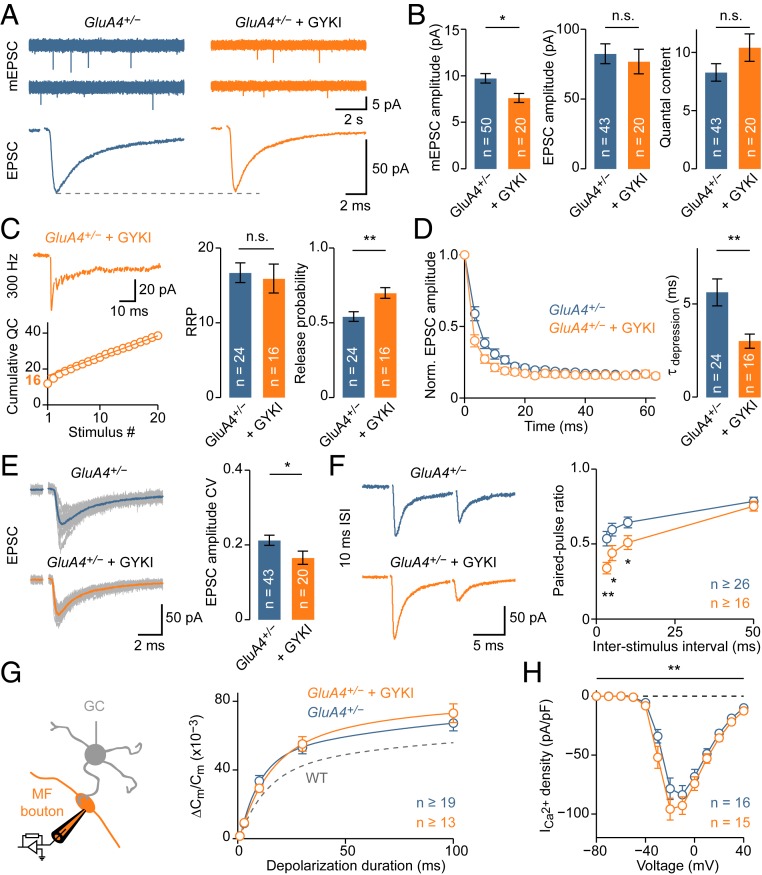
Synergistic homeostatic control of RRP size and release probability. (*A*) Representative mEPSCs and AP-evoked EPSCs in *GluA4*^*+/−*^ mice under control and in the presence of 2 µM GYKI 53655. (*B*) Average mEPSC amplitude (*d* = −0.84; *P* = 0.011), EPSC amplitude (*d* = −0.14; *P* = 0.65), and quantal content (= EPSC/mEPSC; *d* = 0.41; *P* = 0.12). (*C*, *Left*) Representative 300-Hz train. (*C*, *Right*) Average RRP size (*d* = −0.11; *P* = 0.73) and release probability (*d* = 1.04; *P* = 0.003). (*D*, *Left*) Average EPSC amplitude during train stimulation, normalized to the first EPSC. (*D*, *Right*) Average synaptic depression time constant during train stimulation (*d* = −0.90; *P* = 0.0081). Faster depression of GYKI-treated *GluA4*^*+/−*^ synapses suggests increased *p*_r_. (*E*, *Left*) Example EPSCs (24 single responses are overlaid with the average) in *GluA4*^*+/−*^ under control and with GYKI. Same examples as in *A*. (*E*, *Right*) Average data of EPSC amplitude coefficient of variation (CV), which was decreased by GYKI (*d* = −0.58; *P* = 0.04). (*F*, *Left*) Representative EPSC recordings at 10-ms ISI. (*F*, *Right*) Average PPR versus ISI. (*G*, *Left*) Schematic of presynaptic recordings. (*G*, *Right*) Average ∆C_m_ normalized to resting C_m_. Presynaptic recordings reveal no further enhancement of RRP in *GluA4*^*+/−*^ upon GYKI treatment (η^2^ < 0.001; *P* = 0.92; 2-way ANOVA). Dashed line replots WT data from Fig. 3*F*. (*H*) GYKI slightly enhanced I_Ca_ density in *GluA4*^*+/−*^ (η^2^ = 0.02; *P* = 0.004; 2-way ANOVA; post hoc, n.s.).

Next, we investigated PHP after combined pharmacological and genetic AMPAR perturbation. GYKI application (2 µM) to slices of *GluA4*^*+/−*^ mice further reduced mEPSC amplitudes by 22%, but EPSC amplitudes remained similar to baseline values (Fig. 4 *A* and *B* and *SI Appendix*, Fig. S5 *A*–*E*). Accordingly, there was an additional 26% increase in quantal content at *GluA4*^*+/−*^ synapses incubated with GYKI (Fig. 4*B*). High-frequency train stimulation indicated no additional increase in effective RRP size at GYKI-treated *GluA4*^*+/−*^ synapses (Fig. 4*C* and *SI Appendix*, Fig. S5*F*), suggesting that another process enhanced release. Indeed, the stimulus train-based *p*_r_ estimate was significantly increased upon GYKI treatment (Fig. 4*C*). Furthermore, EPSC amplitude CV and PPRs were significantly decreased (Fig. 4 *E* and *F* and *SI Appendix*, Fig. S5*G*), indicating increased *p*_r_. We also observed a correlation between *p*_r_ and mEPSC amplitude reduction at GYKI-treated *GluA4*^*+/−*^ synapses, implying that *p*_r_ potentiation scales with receptor impairment (*SI Appendix*, Fig. S6).

Presynaptic capacitance measurements revealed similar C_m_ jumps between GYKI-treated and GYKI-untreated *GluA4*^*+/−*^ MF boutons (Fig. 4*G*), indicating no further increase in RRP size with respect to WT. Interestingly, there was a small, but significant, increase in Ca^2+^-current density in *GluA4*^*+/−*^ MF boutons treated with GYKI (Fig. 4*H*, see Fig. 6D) without apparent changes in Ca^2+^-current kinetics (*SI Appendix*, Fig. S5 *H* and *I*). This suggests elevated presynaptic Ca^2+^-channel levels at GYKI-treated *GluA4*^*+/−*^ boutons. Given the power relationship between presynaptic Ca^2+^ influx and exocytosis (43), the relatively moderate increase in Ca^2+^-current density is expected to produce a significant increase in *p*_r_, consistent with the enhanced *p*_r_ inferred from postsynaptic recordings (Fig. 4 *C*–*F*). Together, these findings provide evidence that RRP size and *p*_r_ can be synergistically modulated to achieve homeostatic stabilization of synaptic transmission, depending on the magnitude or type of receptor perturbation. By extension, this “sequential” regulation of RRP size and *p*_r_ points toward a potential hierarchy in the modulation of presynaptic function during PHP (*Discussion*).

### GluA4 Is the Major AMPAR Subunit at MF-GC Synapses.

**Fig. 5.**
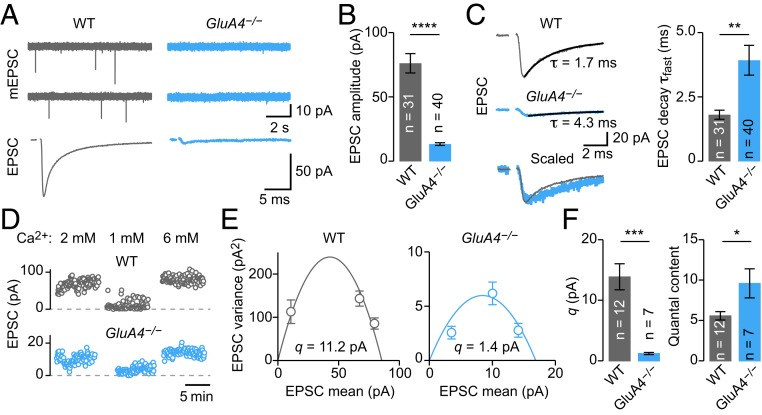
GluA4 is the major AMPAR subunit at MF-GC synapses. (*A*) Representative mEPSCs and AP-evoked EPSCs in WT and *GluA4*^*−/−*^. (*B*) Average EPSC amplitude (*d* = −1.96; *P* = 8.1*e*−14). (*C*, *Left*) Representative EPSCs overlaid with biexponential decay fits. Time constants of the fast component are indicated. (*C*, *Right*) Average fast decay time constant (*d* = 0.84; *P* = 0.0026). (*D*) EPSC amplitudes at indicated extracellular Ca^2+^ concentrations of a representative WT recording (*Upper*) and *GluA4*^*−/−*^ (*Lower*). (*E*) EPSC amplitude variance versus mean for the examples in *D*. Quantal size (*q*) estimates obtained from parabolic fits are indicated. (*F*) Average data from variance-mean analysis. *GluA4*^*−/−*^ synapses have a strongly reduced *q* (*d* = −2.84; *P* = 0.0004). Although EPSC amplitudes are smaller in *GluA4*^*−/−*^ (*d* = −2.1; *P* = 0.0051), quantal content is increased (*d* = 1.01; *P* = 0.017).

Loss of one *GluA4* copy was sufficient to reduce mEPSC amplitudes, indicating an important role for this AMPAR subunit in synaptic transmission at MF-GC synapses. To further elucidate the contribution of GluA4, we recorded from homozygous *GluA4*^*−/−*^ mice that completely lack the GluA4 AMPAR subunit (39). There were almost no detectable spontaneous mEPSCs at *GluA4*^*−/−*^ GCs (Fig. 5*A*), preventing an amplitude quantification. Evoked EPSCs were very small (18% of WT) but could reliably be observed (Fig. 5 *A* and *B*). The EPSC decay time constant was slower than in WT (Fig. 5*C*), owing to the loss of the fast GluA4 subunit (40, 44). Thus, GluA4 is the major AMPAR subunit mediating fast excitation of cerebellar GCs, the most abundant neuronal cell type in the mammalian CNS.

The pronounced reduction in EPSC amplitude at *GluA4*^*−/−*^ MF-GC synapses suggests that PHP is not engaged, saturated, or masked by too strong AMPAR impairment. To distinguish between these possibilities, we first asked if GluA4-lacking synapses express PHP. The small amplitude of mEPSCs (Fig. 5*A*) precluded quantification and direct calculation of quantal content. We therefore used multiple-probability fluctuation analysis to dissect quantal parameters of synaptic transmission (45). We recorded EPSCs under several *p*_r_ conditions by varying the extracellular Ca^2+^ concentration ([Ca^2+^]_e_; Fig. 5*D*) and plotted EPSC amplitude variance against mean (Fig. 5*E*). A parabolic fit to the data provided an estimate for quantal size (*q*), which was 1.2 ± 0.2 pA in *GluA4*^*−/−*^ (9% of WT; Fig. 5*F* and *SI Appendix*, Fig. S7*A*). Using this *q* estimate and the EPSC amplitude at 2 mM [Ca^2+^]_e_, quantal content was strongly increased in *GluA4*^*−/−*^ mutants compared to WT (172% of WT; Fig. 5*F* and *SI Appendix*, Fig. S7 *B*–*D*). Thus, even under strong genetic AMPAR perturbation by ablation of two *GluA4* copies, MF-GC synapses show a prominent increase in neurotransmitter release. Yet, this increase is not sufficient to maintain EPSC amplitudes at WT levels, suggesting that PHP is saturated or masked by too strong receptor impairment.

### Enhanced Exocytosis and Ca^2+^ Influx at *GluA4*^*−/−*^ Boutons.

**Fig. 6.**
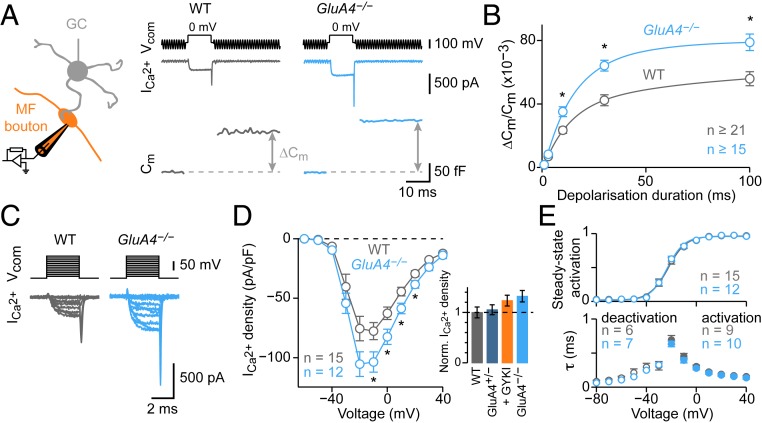
Enhanced exocytosis and Ca^2+^ influx at GluA4-deficient boutons. (*A*, *Left*) Schematic of presynaptic recordings. (*A*, *Right*) Voltage command (V_com_, 10-ms depolarization to 0 mV, *Top*), representative Ca^2+^ currents (I_Ca_, *Middle*), and C_m_ jumps (*Bottom*) for WT and *GluA4*^*−/−*^. (*B*) Average ∆C_m_ versus depolarization duration normalized to resting C_m_ (η^2^ = 0.179; *P* = 2.2*e*−09; 2-way ANOVA). Lines are biexponential fits. (*C*) Representative I_Ca_ recordings. (*D*) I_Ca_ density versus voltage (η^2^ = 0.065; *P* = 3.1*e*−06; 2-way ANOVA). *Inset* shows maximum I_Ca_ density of indicated genotypes for comparison (normalized to WT). (*E*, *Upper*) Average data for steady-state activation; lines are sigmoidal fits. (*E*, *Lower*) Time constants of I_Ca_ activation (filled symbols) and deactivation (open symbols).

So far, the evidence for presynaptic modulation at *GluA4*^*−/−*^ synapses is based on postsynaptic recordings that are likely limited by the strong reduction in AMPAR currents. We therefore used presynaptic recordings (Fig. 6*A*) to directly investigate how neurotransmitter release is modulated at *GluA4*^*−/−*^ boutons, and to ask if PHP is saturated or masked. *GluA4*^*−/−*^ boutons displayed significantly larger C_m_ jumps than WT (Fig. 6*B*), consistent with the increase in quantal content inferred from postsynaptic recordings (Fig. 5*F* and *SI Appendix*, Fig. S7*A*). Moreover, C_m_ jumps were larger in *GluA4*^*−/−*^ animals compared to *GluA4*^*+/−*^ (*SI Appendix*, Fig. S8 *C* and *E*), suggesting a pronounced increase in RRP size that correlates with the degree of *GluA4* loss. However, the increase in ∆C_m_ in *GluA4*^*−/−*^ (∼40%) is at least an order of magnitude smaller than what would be required to maintain WT EPSC levels. We conclude that PHP is saturated under these conditions.

To further explore the mechanisms underlying the increase in exocytosis observed at *GluA4*^*−/−*^ synapses, we recorded presynaptic Ca^2+^ currents at MF boutons (Fig. 6*C*). Ca^2+^-current density of *GluA4*^*−/−*^ boutons was significantly elevated (Fig. 6*D* and *SI Appendix*, Fig. S8*D*). There was no change in steady-state activation or time constants of activation and deactivation in *GluA4*^*−/−*^ mutants (Fig. 6*E*), indicating no major differences in the voltage dependence of Ca^2+^-channel activation or channel kinetics. Presynaptic current-clamp analysis showed similar resting membrane potential, AP amplitude, and AP duration between *GluA4*^*−/−*^ and WT boutons (*SI Appendix*, Fig. S8 *A* and *B*). These data suggest that increased presynaptic Ca^2+^ influx due to elevated Ca^2+^-channel density enhances *p*_r_ in *GluA4*^*−/−*^ mutants. In combination with the increase in ∆C_m_, this indicates a synergistic modulation of RRP size and *p*_r_ in the *GluA4*^*−/−*^ mutant background.

## Discussion

Our results establish that distinct presynaptic homeostatic mechanisms compensate for neurotransmitter receptor impairment on time scales ranging from minutes to months at a mammalian central synapse. In the CNS, presynaptic and postsynaptic forms of homeostatic plasticity are typically observed after inhibition of neuronal activity for hours to days (8–10, 21, 22, 46, 47). Hence, the homeostatic control of neurotransmitter release uncovered here is expressed on a comparably fast time scale, similar to neuromuscular synapses in *Drosophila* and mouse (13, 16). We also demonstrate that acutely induced PHP fully reverses within tens of minutes. In *Drosophila*, there is recent evidence that PHP is reversible within days, although the reversibility time course was likely limited by the nature of receptor perturbation (48). By contrast, PHP reverses within minutes after the removal of pharmacological receptor inhibition at the mouse NMJ (16), similar to our results. The rapid time course of PHP may allow stabilizing information transfer in the CNS on fast time scales. It is an intriguing possibility that PHP serves as a mechanism to compensate for Hebbian plasticity at CNS synapses. In this regard, our work may have implications for neural network simulations incorporating Hebbian plasticity rules, because their stability requires fast compensatory processes with temporal dynamics similar to the form of PHP discovered here (31).

The major presynaptic parameter that was modulated upon AMPAR perturbation in every experimental condition is RRP size. In addition to evidence based on postsynaptic recordings, we directly demonstrate increased RRP size employing presynaptic C_m_ measurements. Importantly, presynaptic RRP measurements are unaffected by potential differences between evoked and spontaneous release (37, 38). Homeostatic regulation of RRP or recycling pool size has been observed at synapses in different species (16, 18, 27, 49, 50), implying a general, evolutionarily conserved mechanism.

In addition to RRP size, we provide evidence for homeostatic *p*_r_ modulation under specific experimental conditions. Homeostatic *p*_r_ changes have been described for the *Drosophila* NMJ (51, 52) and in mammalian CNS cell culture (18, 25, 46, 47), suggesting evolutionary conservation. A major factor linked to homeostatic *p*_r_ control is regulation of presynaptic Ca^2+^ influx (22, 51). Consistently, we uncovered elevated Ca^2+^-current density concomitant with increased *p*_r_, implying increased levels of presynaptic voltage-gated Ca^2+^ channels. This observation is in line with previous light microscopy-based results from mammalian CNS synapses and the *Drosophila* NMJ (21, 52, 53).

We only observed changes in presynaptic Ca^2+^ influx in homozygous *GluA4*^*−/−*^ mutants and after pharmacological receptor perturbation in the *GluA4*^*+/−*^ background. The specific modulation under these experimental conditions may be due to the nature or degree of receptor perturbation. The fact that pharmacological inhibition of WT receptors by a similar degree as in *GluA4*^*+/−*^ mutants induced no apparent changes in *p*_r_ argues against the latter possibility (*SI Appendix*, Fig. S6). Future work is required to explore the links between *p*_r_ modulation and receptor perturbation during PHP.

Whereas RRP size changed in every experimental condition, *p*_r_ modulation was only seen in combination with RRP regulation, suggesting a synergistic, hierarchical modulation of RRP size and *p*_r_ during PHP. There is no coherent picture regarding the relationship between RRP and *p*_r_ regulation during PHP at other synapses. At the *Drosophila* NMJ, genetic data suggests that distinct molecular pathways underlie homeostatic modulation of RRP and Ca^2+^ influx/*p*_r_ (50). There is recent evidence that homeostatic vesicle pool regulation requires altered Ca^2+^ influx through P/Q-type Ca^2+^ channels at cultured mammalian synapses (22). By contrast, PHP at the mouse NMJ involves RRP modulation without any evidence for changes in *p*_r_ (16). Although future experiments are required to relate these observations, this points toward the possibility that different synapses may engage different presynaptic mechanisms during PHP.

A major finding of this study is that synaptic transmission at cerebellar MF-GC synapses strongly depends on the GluA4 AMPAR subunit. The GluA4 subunit, encoded by *GRIA4*, confers rapid kinetics and large conductance to mammalian AMPARs (42). Previous work at the calyx of Held synapse demonstrated a role of GluA4 for fast sensory processing and indicated partial presynaptic compensation in *GluA4*^*−/−*^ mice (44). Cerebellar mossy fibers are capable of firing at remarkably high frequencies (33), and our data show that GluA4 is the predominant AMPAR subunit at MF-GC synapses. These findings suggest that different CNS synapses rely on the GluA4 subunit for rapid information transfer. Mutations in *GRIA4* have been implicated in schizophrenia and intellectual disability (54, 55), underscoring the importance of this AMPAR subunit for synaptic function.

Our findings advance our understanding of homeostatic plasticity by revealing rapid and reversible regulation of specific presynaptic mechanisms upon glutamate receptor perturbation at a central synapse. In addition, we demonstrate a conserved process between presynaptic homeostatic plasticity studied at the *Drosophila* NMJ and the maintenance of neural function in the mammalian CNS. It will be exciting to explore the underlying molecular mechanisms and the interface between PHP and other forms of synaptic plasticity in the CNS (30, 31).

## Methods

Full methods are available in *SI Appendix*.

### Animals.

Animals were treated in accordance with national and institutional guidelines. All experiments were approved by the Cantonal Veterinary Office of Zurich (authorization no. ZH206/16). Recordings were made from adult (3- to 10-wk-old) C57BL/6JRj mice or *GRIA4* knockout mice (ref. 39; termed “*GluA4*^*+/−*^” and “*GluA4*^*−/−*^” throughout the manuscript).

### Slice Electrophysiology.

Cerebellar slices were prepared as described previously (33, 56). The ACSF contained (in mM): 125 NaCl, 25 NaHCO_3_, 20 glucose, 2.5 KCl, 2 CaCl_2_, 1.25 NaH_2_PO_4_, 1 MgCl_2_, equilibrated with 95% O_2_ and 5% CO_2_, pH 7.3, ∼310 mOsm. Whole-cell recordings from MF boutons and GCs were performed at room temperature using an EPC10 amplifier (HEKA Elektronik) as described previously (33, 56).

### Statistical Analysis.

Significance of datasets was examined using 2-sided unpaired or paired Student *t* tests, or 2-way analysis of variance (ANOVA). *P < 0.05; **P < 0.01; ***P < 0.001; ****P < 0.0001; n.s. not significant. Data are presented as mean ± SEM.

### Data Availability Statement.

All data discussed in the paper will be made available to readers.

## Supplementary Material

Supplementary File
