# Supplementary material for: Rapid and sustained homeostatic control of presynaptic exocytosis at a central synapse
Source: Proc Natl Acad Sci U S A. 2019 Nov 4;116(47):23783–9. doi: 10.1073/pnas.1909675116 (PMC6876255; doi:10.1073/pnas.1909675116)
Supplement: Supplementary File [file pnas.1909675116.sapp.pdf]

PNAS
www.pnas.org

Supplementary Information for

**Rapid and Sustained Homeostatic Control of Presynaptic Exocytosis at a Central Synapse**

Igor Delvendahl, Katarzyna Kita, and Martin Müller

Corresponding author: Martin Müller
Email: martin.mueller@imls.uzh.ch

**This PDF file includes:**

Supplementary Methods
Figures S1 to S8
Tables S1 to S3
SI References



www.pnas.org/cgi/doi/10.1073/pnas.1909675116

**Supplementary Methods**

**Animals.** Animals were treated in accordance with national and institutional guidelines. All experiments were approved by the Cantonal Veterinary Office of Zurich (authorization no. ZH206/16). Wild-type C57BL/6JRj mice were obtained from Janvier Labs; *GRIA4* knock-out mice were a kind gift of H. Monyer ((1); termed "*GluA4*$^{+/-}$" and "*GluA4*$^{-/-}$" throughout the manuscript). *GluA4*$^{-/-}$, *GluA4*$^{+/-}$, and wild-type (WT) littermates were bred from heterozygous crosses. Adult (3- to 10-wk-old) mice of either sex were used for experiments. All animals were maintained with food ad libitum on a 12h/12h light/dark cycle, and experiments were performed between 9 AM and 9 PM.

Genotyping of *GRIA4* mice was performed by PCR analysis of genomic DNA from toe biopsies. For genotyping, the forward primer 5'-CGTGCGCCACCACCGCCCGG-3' was used with the reverse primer 5'-TGCCACTCAGTTATTGCATCAC-3' to detect a 290 bp band in wild-type, and with the reverse primer 5'-CAAAACATGGATTAGTCTTTATGGAACAG-3' to detect a 500 bp band in *GRIA4* mutant mice.

**Slice Electrophysiology.** Mice were sacrificed by rapid decapitation; the cerebellar vermis was removed quickly and mounted in a chamber filled with cooled extracellular solution. 300-µm thin parasagittal slices were cut using a Leica VT1200S vibratome (Leica Microsystems), transferred to an incubation chamber at ~35 °C for 30 min and then stored at room temperature until experiments. The extracellular solution (artificial cerebrospinal fluid, ACSF) for slice cutting and storage contained (in mM): 125 NaCl, 25 $NaHCO_3$, 20 glucose, 2.5 KCl, 2 $CaCl_2$, 1.25 $NaH_2PO_4$, 1 $MgCl_2$, equilibrated with 95% $O_2$ and 5% $CO_2$, pH 7.3, ~310 mOsm. Chemicals were obtained from Sigma-Aldrich unless otherwise stated.

**Postsynaptic Recordings.** Slices were visualized using an upright microscope (Scientifica) with a 60×, 1 NA water-immersion objective, infrared optics, and differential interference contrast. Cerebellar granule cells were identified as described previously (2, 3). Recordings were performed in lobules III–VI of the cerebellar vermis. The recording chamber was continuously perfused with ACSF supplemented with 10 µM bicuculline to block $GABA_A$ receptors, 1 µM strychnine to block glycine receptors, and 10 µM D-APV to block NMDA receptors. In a subset of recordings, the ACSF contained 50 µM cyclothiazide (CTZ; final DMSO concentration, 0.05%) and 1 mM kynurenic acid (Kyn) to prevent postsynaptic AMPA receptor desensitization and saturation, respectively. In some recordings, D-APV was omitted, or bicuculline was replaced by 50 µM picrotoxin (final DMSO concentration, 0.05%).

Postsynaptic patch pipettes were pulled to open-tip resistances of 5–9 MΩ (when filled with intracellular solution) from 1.5 mm/1.05 mm (OD/ID) borosilicate glass (Science Products) using a Sutter P-97 horizontal puller (Sutter Instruments). The intracellular solution contained (in mM): 150 K-gluconate, 10 NaCl, 10 HEPES, 3 MgATP, 0.3 NaGTP, 0.05 ethyleneglycol-bis(2-aminoethylether)-N,N,N',N'-tetraacetic acid (EGTA), pH adjusted to 7.3 using KOH. Voltages were corrected for a liquid junction potential of +13 mV.

**EPSC recordings.** Whole-cell recordings were performed at room temperature (21–25 °C) using an EPC10 amplifier (HEKA Elektronik). Voltage-clamp recordings were filtered with the internal low-pass filter of the amplifier at 10 kHz and digitized at 100–200 kHz; holding potential was −80 mV. Postsynaptic series resistance was typically <30 MΩ (median 22.9, range 12–50) and not compensated. Extracellular mossy fiber stimulation was performed using bipolar square voltage pulses (duration, 150 µs) generated by an ISO-STIM 01B stimulus isolation unit (NPI) and applied through an ACSF-filled pipette. The pipette was moved over the slice surface in the

vicinity of the postsynaptic cell while applying voltage pulses until excitatory postsynaptic currents (EPSCs) could be evoked reliably. Care was taken to stimulate single mossy fiber inputs, as demonstrated by robust average EPSC amplitudes when increasing stimulation intensity (4). Stimulation was performed at 1–2 V above the threshold, typically <15 V.

EPSCs were recorded at a stimulation frequency of 0.1 Hz. The decay of EPSCs was fit with a biexponential function (5, 6) constrained to reach the baseline before stimulation onset. Under all recording conditions and in all genotypes, the MF-GC EPSC decay could be well described by a biexponential function (>20% contribution of $\tau$ slow). EPSC recordings during high-frequency train stimulation were performed as described previously (5). Trains comprising 20 stimuli at 300 Hz were applied every 30 s. EPSC amplitudes during the train were quantified as the difference between the peak EPSC amplitude and the baseline current right after stimulation (5). The number of release-ready vesicles was estimated by back-extrapolation of the cumulative EPSC amplitude as described previously (7): A line was fit to the last 10 pulses of the cumulative EPSC amplitudes of the 300-Hz train. Extrapolation of the linear fit to t = 0 and division by the average mEPSC amplitude yielded an estimate of the initial number of release-ready vesicles (readily releasable pool; RRP) before onset of stimulation. For paired-pulse ratios (PPR), paired pulses with inter-stimulus intervals of 3.33–50 ms were applied at 0.1 Hz. EPSCs from five consecutive sweeps for a given ISI were averaged and PPR was calculated as the ratio of second over first average EPSC.

Spontaneous miniature EPSCs (mEPSCs) were recorded from a holding potential of −80 mV or −100 mV, filtered at 2.9 kHz and digitized at 50 kHz. Amplitudes of mEPSCs recorded at −100 mV were scaled down for quantal content calculation according to the linear current-voltage relation of AMPA receptors at MF-GC synapses with a reversal potential of ~0 mV (4, 8). We confirmed the linear current-voltage relation of EPSCs and the reversal potential under our recording conditions. Data were recorded for 60–240 s and mEPSCs were detected with a template matching algorithm implemented in Neuromatic (9) running in Igor Pro (Wavemetrics). The average mEPSC amplitude was calculated from all detected events in a recording after visual inspection for false positives.

To investigate the effect of sub-saturating AMPAR block, 2 µM of GYKI 53655 was added to the ACSF. After different incubation times, whole-cell recordings were obtained from GCs in the continued presence of GYKI; times given in figures refer to incubation time. To study the reversibility of PHP, slices were incubated in 2 µM GYKI for 30 min and subsequently exposed to control ACSF. Times indicated in figures refer to the time of control ACSF exposure before whole-cell recordings were begun. Data in Figure 1F includes some recordings during which washout of GYKI was performed during whole-cell recording.

**Variance-Mean Analysis.** For variance-mean analysis, EPSCs were recorded at −80 mV (corrected for the liquid junction potential) using an intracellular solution containing (in mM): 135 Cs-gluconate, 20 TEA-Cl,10 HEPES, 5 $Na_2$phosphocreatine, 4 MgATP, 2 QX-314, 0.3 NaGTP, 0.2 EGTA, pH adjusted to 7.3 using CsOH. Release probability was altered by varying extracellular $Ca^{2+}$ concentration. The ACSF contained the following combinations of $Ca^{2+}$/$Mg^{2+}$: 2/1, 1/5, 4/1, 6/0.5. A total of 50–100 EPSCs were recorded per $Ca^{2+}$ concentration at a frequency of 0.2 Hz. Only cells where data from at least three $Ca^{2+}$ concentrations could be obtained were included for analysis. EPSC amplitudes were calculated from a 100 µs time window centered around the peak; baseline variance was determined in a 100 µs time window preceding the stimulation (10). The variance of EPSC amplitudes was calculated as:

$$\sigma^2 = \frac{1}{n}\sum_{i=1}^{n}(I_i - \bar{I})^2 \qquad \text{(Eq. 1)}$$

The variance of sample variance was calculated as:

$$\sigma^2 = \frac{n}{(n-2)(n-3)}\left(\frac{3(3-2n)(n-1)^2 - n(n-2)(n-3)^2}{(n^2-2n+3)(n-1)^2}\, m_2^2 + m_4\right) \qquad \text{(Eq. 2)}$$

where $m_2$ and $m_4$ are the central moments calculated as:

$$m_r = \frac{1}{n}\sum_{i=1}^{n}(X_i - \bar{X})^r \qquad \text{(Eq. 3) (11)}$$

Variance was then plotted against mean EPSC amplitude for all $Ca^{2+}$ concentrations and fit by:

$$\sigma^2(I) = q\,I - \frac{I^2}{N} \qquad \text{(Eq. 4)}$$

where $I$ is the mean EPSC amplitude and $q$ and $N$ represent quantal size and the number of functional release sites (12). Fits were weighted by the inverse SEM.

To calculate quantal size in *GluA4*$^{-/-}$ mice, two additional approaches were employed: (i) $q$ was calculated from the limiting slope of the variance-mean plot (n = 16 for *GluA4*$^{-/-}$ and n = 11 for WT). Using this $q$ estimate and the average EPSC amplitude from a larger set of recordings (n = 39 for *GluA4*$^{-/-}$ and n = 31 for WT, Fig. 3B), mean quantal content was calculated using bootstrap procedures (100,000 resamples for each, $q$ and EPSC amplitude, (13)). (ii) $q$ was calculated from $1/CV^2$ analysis of EPSC recordings at low $Ca^{2+}$ concentration (i.e., 1 mM). At low release probability ($p_r$), $1/CV^2$ equals quantal content and $q$ can be calculated from the average EPSC amplitude accordingly (14). The three estimates of $q$ were in good agreement (cf. *SI Appendix* Fig. S7).

**Single-channel conductance.** Single-channel conductance of AMPARs at MF-GC synapses was determined from the variance of peak-scaled mEPSC decays (15). Recordings with at least 40 events were included for analysis. Individual mEPSCs were peak-scaled to the average and subsequently subtracted from the average. Variance was calculated from the difference traces in 100 bins of equal fractional amplitude reduction within the decay phase of mEPSCs. Variance was plotted against mean amplitude and the initial 75% of the data were fit with a parabola (Eq. 4), yielding the single-channel conductance (15).

**Presynaptic Recordings.** Cerebellar mossy fiber boutons were identified based upon morphology and passive membrane parameters as described previously (3, 16, 17). Recordings were performed in lobules III–VI of the cerebellar vermis. For current-clamp recordings, the same solutions as for postsynaptic recordings were used (ACSF, K-gluconate-based internal). Presynaptic APs were recorded and analyzed as described previously (3). For voltage-clamp measurements, the slice recording chamber was continuously perfused with ACSF containing (in mM): 105 NaCl, 25 $NaHCO_3$, 25 glucose, 20 TEA-Cl, 5 4-AP, 2.5 KCl, 2 $CaCl_2$, 1.25 $NaH_2PO_4$, 1 $MgCl_2$, and 0.001 tetrodotoxin (TTX), equilibrated with 95% $O_2$ and 5% $CO_2$. Presynaptic patch pipettes were pulled to open-tip resistances of 5–9 MΩ (when filled with intracellular solution). The intracellular solution contained (in mM): 135 Cs-gluconate, 20 TEA-Cl, 10 HEPES, 5 $Na_2$phosphocreatine, 4 MgATP, 0.3 NaGTP, 0.2 EGTA, pH adjusted to 7.3 using CsOH. Voltages were corrected for a liquid junction potential of +13 mV.

Direct presynaptic whole-cell recordings were performed at room temperature (21–25 °C). Data were filtered with the internal low-pass filter of the amplifier at 10 kHz and digitized at 200 kHz. Presynaptic series resistance was typically <30 MΩ (median 25.9, range 13–53) and compensated online by 40–60% with 10 µs delay. We pharmacologically isolated presynaptic $Ca^{2+}$ currents as previously described (3, 16, 17). $Ca^{2+}$ currents were elicited by square pulses of varying durations and were corrected for leak currents and capacitance currents using the P/4 method. To investigate activation kinetics of presynaptic $Ca^{2+}$ currents, voltage steps of 3 ms duration to varying potentials from a holding potential of −80 mV were used. Steps to varying potentials after full activation (0 mV for 3 ms) were used to study the deactivation of $Ca^{2+}$

currents. Activation and deactivation kinetics were fit and analyzed as described previously (3, 16). Analysis of $Ca^{2+}$-current kinetics was restricted to recordings with series resistance <30 MΩ.

**Membrane capacitance measurements.** Membrane capacitance ($C_m$) measurements were performed using the 'sine + DC' mode (18) of the Patchmaster software lock-in extension as described previously (3, 16, 19). For $C_m$ recordings, a sine-wave with 1 kHz frequency and ±50 mV peak amplitude was superimposed on a holding potential of −100 mV. Resting capacitance was estimated to be 4.6 ± 0.3 pF (for WT; n = 23; median 3.3 pF). In between sine-wave stimulation, the presynaptic terminal was depolarized from −80 mV to 0 mV for 1–100 ms. Hydrostatic pipette pressure during $C_m$ recordings was kept to a low and constant level (20). The $C_m$ increase was determined as the difference between the mean capacitance 50–100 ms after the depolarizing pulse and the baseline during 200 ms before the onset of the depolarizing pulse.

**Statistical Analysis.** Data were analyzed using custom-written routines in Igor Pro software (Wavemetrics). In the figures, stimulation artifacts are blanked for clarity and some examples are digitally filtered to 7.9 kHz using the finite-response filter function in Igor Pro. Figure legends and text state the number of independent cells (n). Statistical testing was performed in R (21). Significance of datasets was examined using two-sided unpaired or paired Student's t tests. Paired-pulse ratios, $\Delta C_m$ data, and $Ca^{2+}$-current densities were tested using 2-way analysis of variance (ANOVA) followed by post-hoc two-sided Student's *t* tests with Bonferroni-Holm correction. Statistical significance is indicated as p values in the figure legends. Effect sizes were calculated as Cohen's *d* (for t-tests) or partial $\eta^2$ (for ANOVA) using the *effsize* and *sjstats* packages in R.

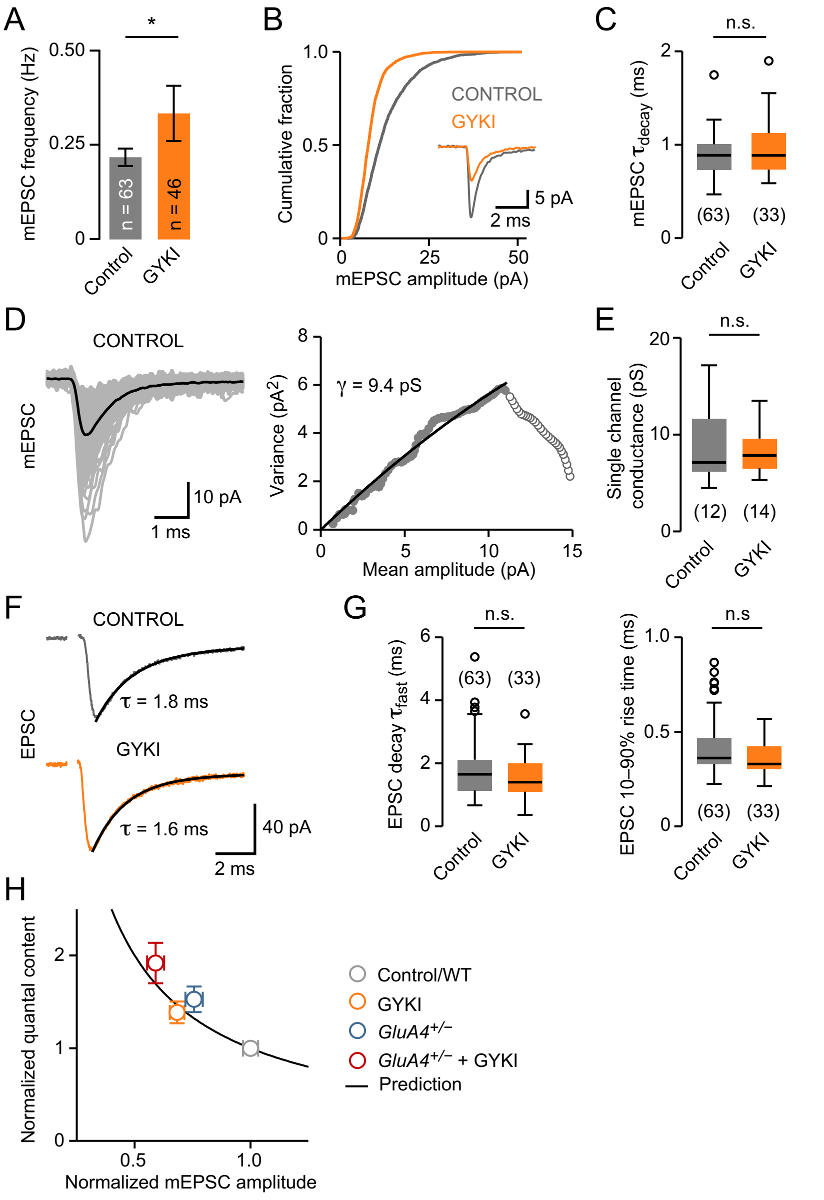


**Fig. S1.** Miniature and Evoked EPSCs after Pharmacological AMPAR Perturbation.

(A) mEPSC frequency for control and GYKI (>20-min incubation; Cohen's $d = 0.45$; $p = 0.037$). (B) Normalized cumulative frequency of mEPSC amplitudes for control and GYKI. Inset shows mEPSC averages from representative cells. (C) Median mEPSC decay time constant for control and GYKI ($d = 0.30$; $p = 0.17$). (D) *Left*: Representative mEPSCs (gray) recorded from a control cell, aligned and overlaid with the average (black). *Right*: Plot of mEPSC decay variance versus mean mEPSC amplitude (15) of the example on the left. A fit to the initial 75% of the data provides an estimate of the single-channel conductance (indicated) (8, 15). (E) Single-channel conductance for control and GYKI. GYKI did not alter single-channel conductance ($d = -0.17$; $p = 0.66$). (F) Representative AP-evoked EPSCs with bi-exponential fits to the decay time course for control and GYKI. Time constants of the fast decay component are indicated. (G) *Left*: Fast decay time constant of evoked EPSCs for both conditions ($d = -0.28$; $p = 0.19$). *Right*: 10–90% EPSC rise times ($d = -0.41$; $p = 0.06$). (H) Normalized quantal content versus normalized

mEPSC amplitude for the experiments from Figs. 1, 3, and 4. Data were normalized to the combined average of control (i.e. without GYKI) and WT. Solid line represents the theoretical prediction of the quantal content required to sustain the mean WT/control EPSC amplitude over a range of mEPSC amplitudes ($f(x) = 1/x$). * $p < 0.05$; n.s. not significant; two-tailed Student's t-test. Bars represent mean ± SEM. Boxplots show median and $1^{st}/3^{rd}$ quartiles, whiskers extend to data within 1.5× the interquartile range from lower or upper quartiles.

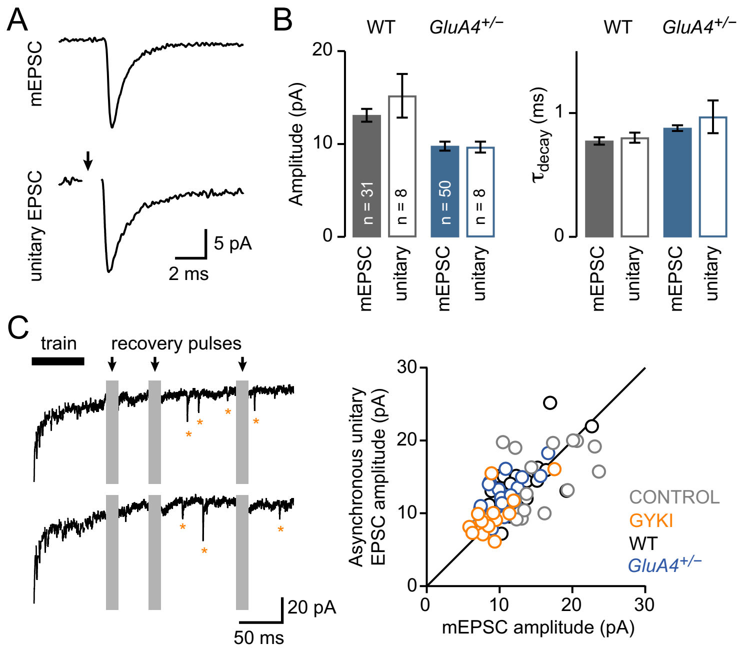


**Fig. S2.** Miniature, Unitary, and Asynchronous, Unitary EPSCs Have Similar Amplitudes.

(A) Examples of mEPSCs (*Top*, average of 16 events) and unitary evoked EPSCs recorded at 1 mM $[Ca^{2+}]_e$ (*Bottom*, average of 16 sweeps). Arrow indicates time of stimulation; stimulation artifact is blanked for clarity. (B) Average (± SEM) amplitude and decay time constant of mEPSCs and unitary EPSCs for WT and *GluA4*$^{+/-}$. Note the similar amplitudes and decay kinetics of mEPSCs and unitary EPSCs, implying that spontaneous and AP-evoked synaptic transmission activate overlapping receptor populations. (C) *Left*: Examples of asynchronous, unitary EPSCs after 300-Hz train stimulation. Events are marked by asterisks, recovery pulses after the train are blanked. *Right*: Average asynchronous unitary EPSC amplitude of the indicated experimental conditions plotted against the average mEPSC amplitude measured in the same cell. Black line represents unity. The correlation between asynchronous unitary EPSC amplitudes and mEPSC amplitudes indicates overlapping receptor populations.

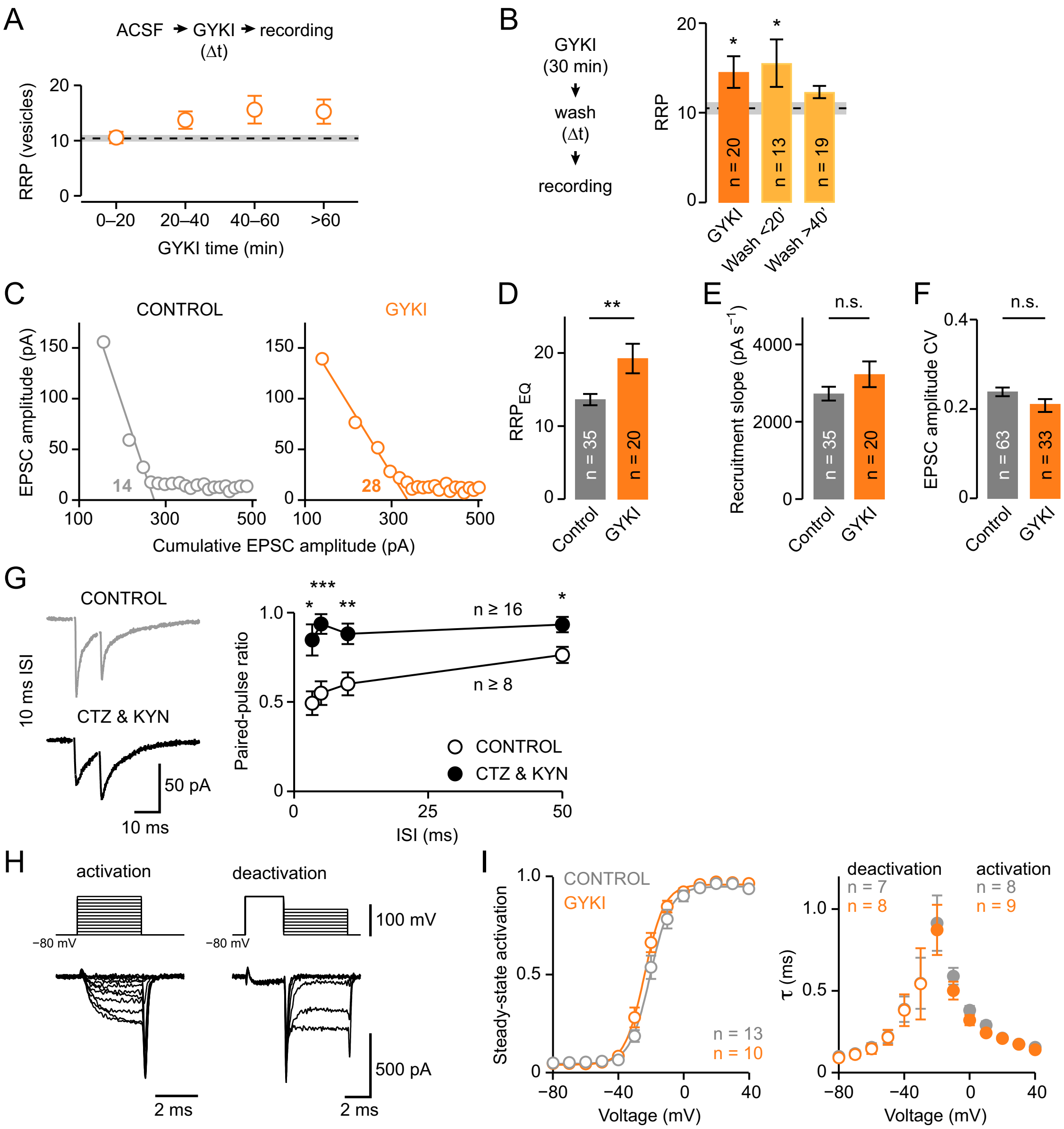


**Fig. S3.** Homeostatic RRP Modulation at MF-GC Synapses.

(A) Average effective readily-releasable vesicle pool (RRP) size estimated from cumulative EPSC amplitude analysis (7) for different durations of GYKI incubation. Dashed lines and shaded area indicate control average and ± SEM. The increase in RRP size parallels the increase in quantal content (cf. Figure 1C). (B) Average RRP size following GYKI wash-out grouped according to wash-out time. Note that RRP size remained increased during early wash-out and returned to control levels after >40 min (cf. Figure 1F). (C) Estimation of RRP size according to Elmqvist and Quastel ($RRP_{EQ}$, (22)). Representative plots of EPSC amplitude versus cumulative EPSC amplitude for 300-Hz train stimulation under control conditions and after GYKI treatment (same recordings as in Figure 2A). Lines are fits to the first four data points; estimated $RRP_{EQ}$ size is indicated [calculated as: (cumulative EPSC)/(mEPSC amplitude)]. (D) Average $RRP_{EQ}$ size for control and GYKI ($d = 0.86$; $p = 0.003$). (E) Average slope of the line fit to cumulative EPSCs (“recruitment slope”) for control and GYKI. (F) Average EPSC amplitude coefficient of variation

[CV = SD/(average EPSC); *d* = −0.39; p = 0.075]. (G) *Left:* Representative EPSCs induced by paired-pulse stimulation (inter-stimulus interval, ISI = 10 ms) recorded in the presence of 50 μM cyclothiazide (CTZ) and 1 mM kynurenic acid (KYN) to minimize AMPAR desensitization and saturation (23). *Right*: Average paired-pulse ratio for different ISIs. CTZ and KYN increased PPRs (p = 1.4E−08; ANOVA), as described previously (23). (H) Analysis of $Ca^{2+}$-channel kinetics. *Top:* Voltage-clamp protocols to measure $Ca^{2+}$-channel activation and deactivation. *Bottom:* Example $Ca^{2+}$-current recordings. (I) *Left:* Average data for steady-state activation calculated from tail currents. Lines are sigmoidal fits. *Right:* Time constants of $Ca^{2+}$-channel activation and deactivation. $Ca^{2+}$-current data were analyzed as described in (3). * p < 0.05; ** p < 0.01; *** p < 0.001; n.s. not significant; two-tailed Student's t-test. Data are mean ± SEM.

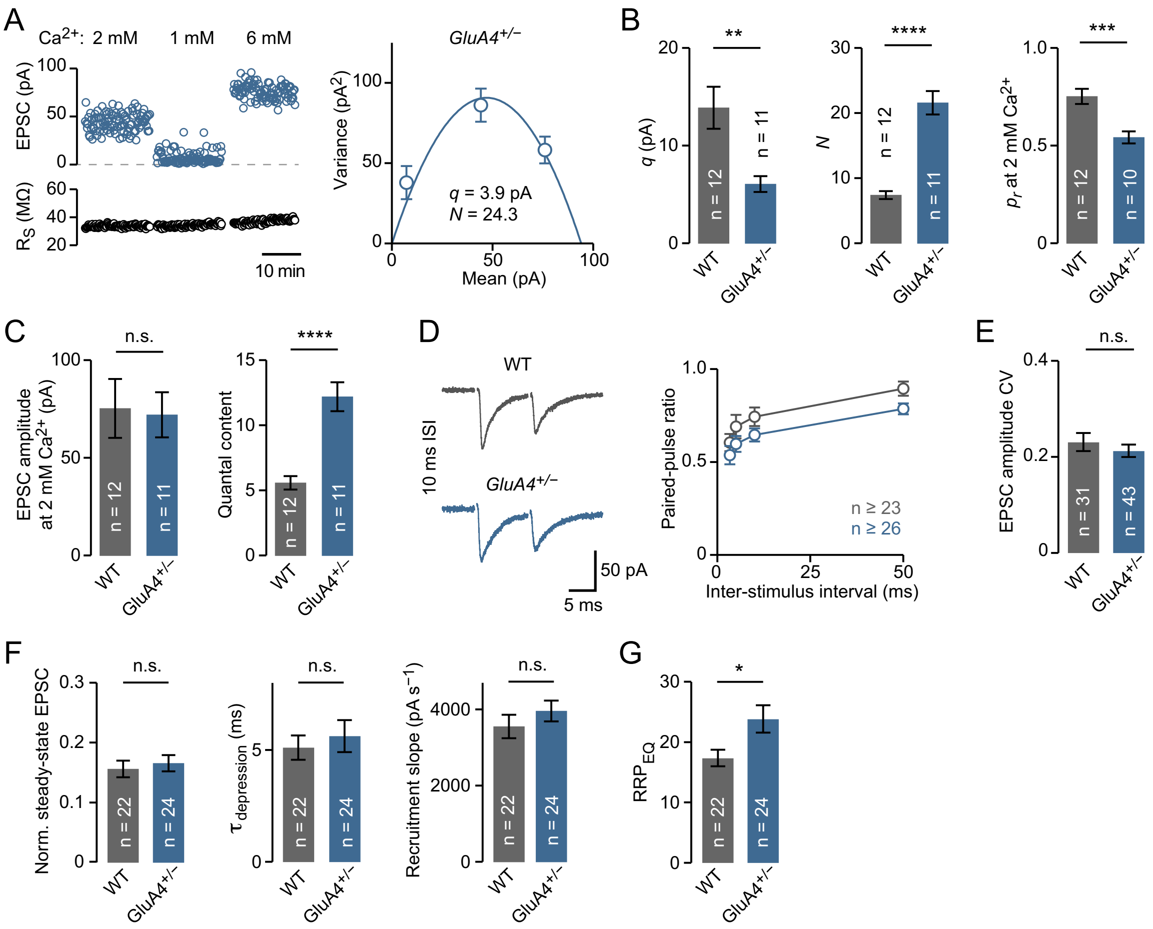


**Fig. S4.** Genetic AMPAR Perturbation Increases Quantal Content through RRP Modulation.

(A) *Left*: EPSC amplitudes recorded at a *GluA4*$^{+/-}$ synapse at different extracellular $Ca^{2+}$ concentrations. *Bottom*: Corresponding series resistance ($R_S$). *Right*: EPSC amplitude variance versus mean of the same synapse. Data were fit with equation 4 (*Methods*); quantal parameters obtained from the fit are indicated. (B) Average data for $q$, $N$ and $p_r$ at 2 mM $Ca^{2+}$ for *GluA4*$^{+/-}$ and WT synapses ($q$: $d = -1.36$; p = 0.004; $N$: $d = 3.23$; p = 1.4E−07; $p_r$: $d = -1.77$; p = 5.2E−04). (C) Average data of EPSC amplitude and quantal content at 2 mM $Ca^{2+}$ for *GluA4*$^{+/-}$ and WT synapses (EPSC: $d = -0.07$; p = 0.87; QC: $d = 2.31$; p = 1.17E−05). (D) *Left*: Example EPSCs induced by paired-pulse stimulation (inter-stimulus interval, ISI = 10 ms). *Right*: Average paired-pulse ratios as a function of ISI for WT and *GluA4*$^{+/-}$ (p = 0.004; ANOVA; post hoc tests: n.s.). (E) Average data for EPSC amplitude coefficient of variation [CV = SD/(average EPSC); $d = -0.19$; p = 0.42]. The similar CV is consistent with unchanged release probability in *GluA4*$^{+/-}$ (cf. Figure S5G). (F) Average steady-state amplitude ($d = 0.15$; p = 0.62), time constant of synaptic depression ($d = 0.17$; p = 0.58), and recruitment slope ($d = 0.41$; p = 0.15) for 300-Hz train stimulation in WT and *GluA4*$^{+/-}$. (G) Average $RRP_{EQ}$ size (22) calculated for WT and *GluA4*$^{+/-}$ ($d = 0.72$; p = 0.02). * $p < 0.05$; ** $p < 0.01$; *** $p < 0.001$; **** $p < 0.0001$; n.s. not significant; two-tailed Student's t-test. Data are mean ± SEM.

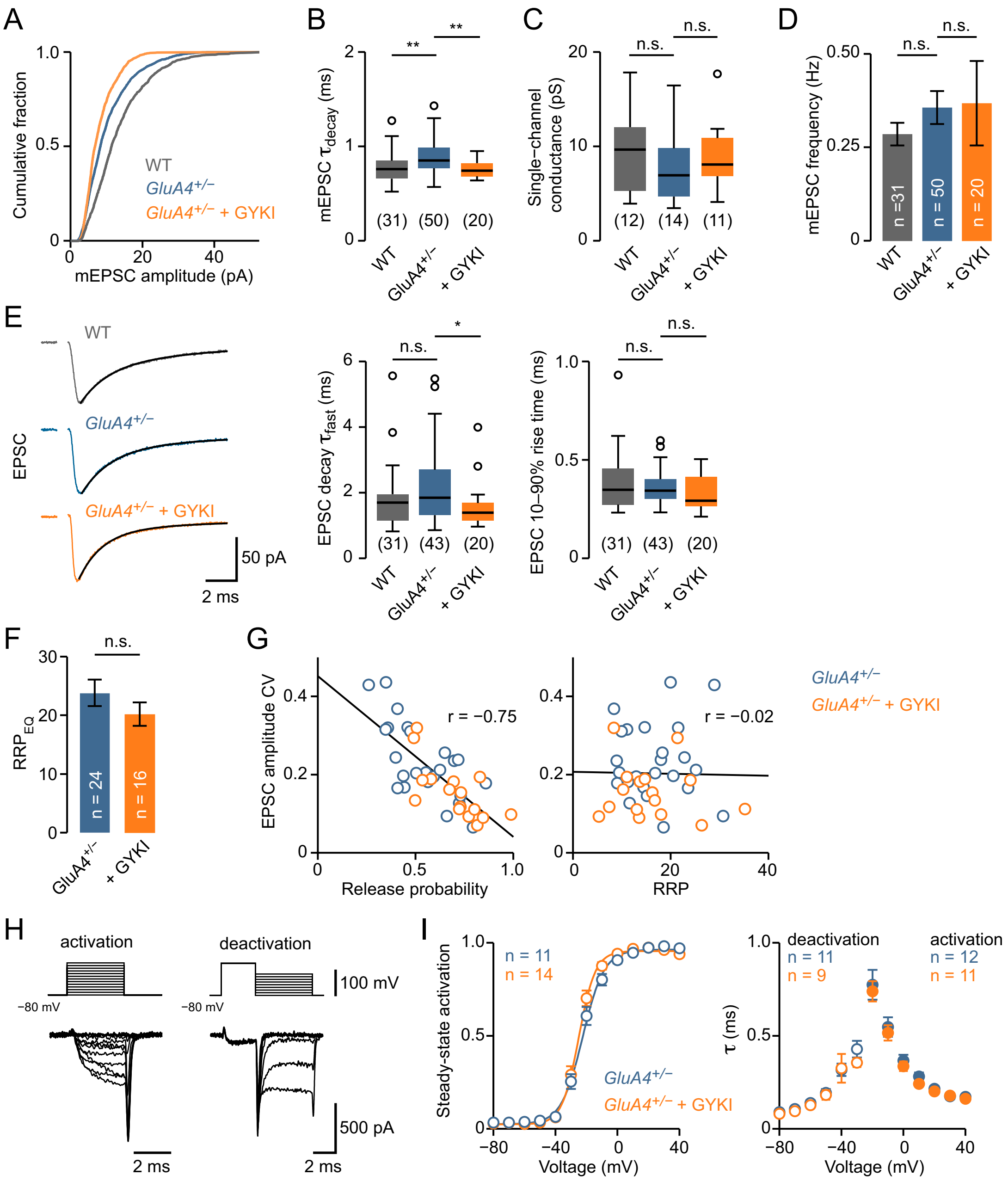


**Fig. S5.** Combined Genetic and Pharmacological AMPAR Perturbation.

(A) Normalized cumulative frequency of mEPSC amplitudes for WT, *GluA4*$^{+/-}$, and *GluA4*$^{+/-}$ with GYKI. (B) mEPSC decay time constant for WT, *GluA4*$^{+/-}$, and *GluA4*$^{+/-}$ with GYKI ($d = 0.65$; $p = 0.006$; $d = -0.97$; $p = 0.005$). (C) Single-channel conductance estimated from mEPSC decay variance-mean data ((15), Fig. S1D) for WT, *GluA4*$^{+/-}$, and *GluA4*$^{+/-}$ with GYKI ($d = -0.24$; $p = 0.55$; $d = 0.21$; $p = 0.62$). (D) Average mEPSC frequency for WT, *GluA4*$^{+/-}$, and *GluA4*$^{+/-}$ with GYKI (WT vs. *GluA4*$^{+/-}$: $d = 0.24$; $p = 0.25$; *GluA4*$^{+/-}$ *vs. GluA4*$^{+/-}$ with GYKI: $d = 0.03$; $p = 0.91$). (E) *Left*: Examples of evoked EPSC recordings. Black lines are bi-exponential fits. *Center*: Fast decay time constant of EPSCs for WT, *GluA4*$^{+/-}$, and *GluA4*$^{+/-}$ with GYKI ($d = 0.38$; $p = 0.13$; $d = -0.72$; $p = 0.031$). *Right*: 10–90% EPSC rise times ($d = -0.17$; $p = 0.45$; $d = -0.36$; $p = 0.19$). (F) Average $RRP_{EQ}$ size (22) for *GluA4*$^{+/-}$ and *GluA4*$^{+/-}$ with GYKI ($d = -0.90$; $p = 0.0081$). (G)

EPSC amplitude coefficient of variation (CV) versus release probability estimated from 300-Hz train stimulation (*Left*; p = 1.9E−08), or versus RRP size (*Right*; p = 0.91). Pearson correlation coefficients are indicated. (H) Analysis of $Ca^{2+}$-channel kinetics. *Top:* Voltage-clamp protocols to measure $Ca^{2+}$-channel activation and deactivation. *Bottom:* Example $Ca^{2+}$-current recordings. (I) *Left*: Average data for steady-state activation calculated from tail currents. Lines are sigmoidal fits. *Right*: Time constants of $Ca^{2+}$-channel activation and deactivation. $Ca^{2+}$-current data were analyzed as described in (3). * $p < 0.05$; ** $p < 0.01$; n.s. not significant; two-tailed Student's t-test. Boxplots show median and $1^{st}/3^{rd}$ quartiles, whiskers extend to data within 1.5× the interquartile range from lower or upper quartiles. Bar graphs show mean ± SEM.

A

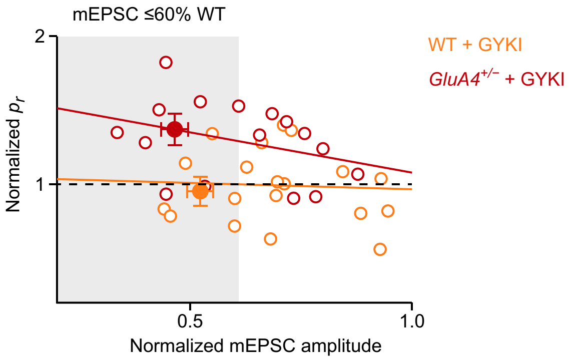


**Fig. S6.** *$P_r$* Modulation Following Pharmacological AMPAR Perturbation.

(A) Normalized release probability ($p_r$) estimated from 300-Hz train stimulation versus normalized mEPSC amplitude. Data are normalized to average of WT/control; solid lines are linear fits. At *GluA4*$^{+/-}$ synapses, $p_r$ was increased by GYKI treatment (in addition to RRP size, cf. Figure 4C), and a correlation between $p_r$ and mEPSC amplitude was observed. At WT synapses, however, GYKI application caused no apparent changes in $p_r$, but an increase in RRP size (cf. Figure 2B). Filled symbols represent average data (± SEM) for WT with GYKI and *GluA4*$^{+/-}$ with GYKI of all cells with mEPSC amplitudes ≤60% of WT/control. Comparing cells with similar relative mEPSC reduction reveals a differential modulation of $p_r$ by GYKI application in *GluA4*$^{+/-}$ and WT.

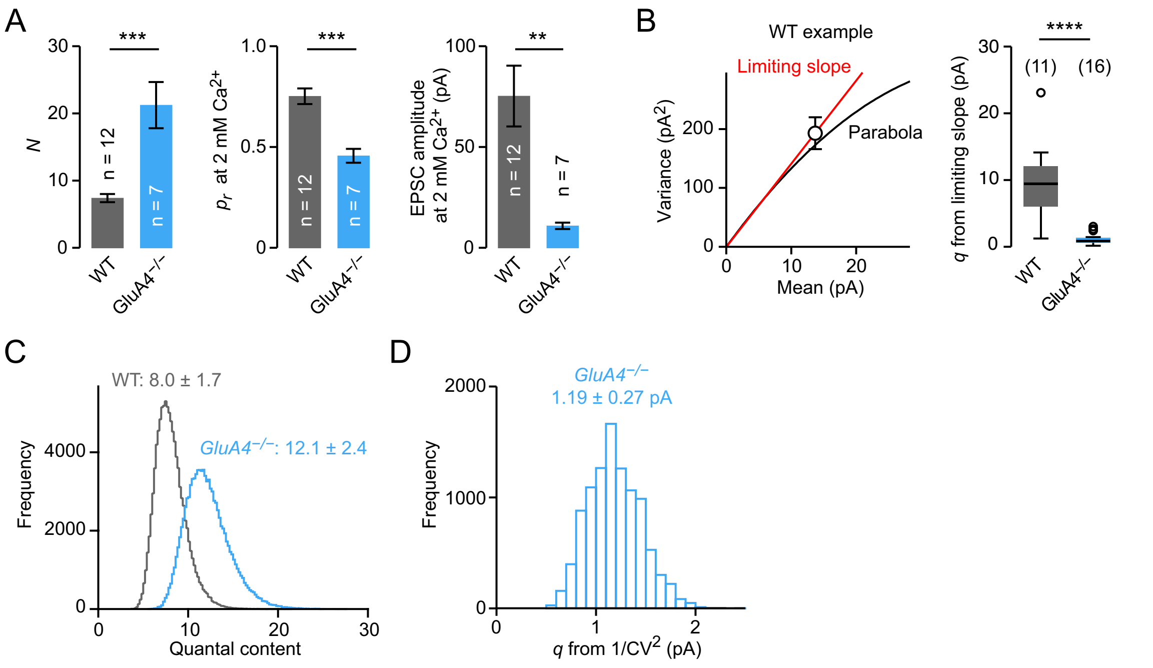


**Fig. S7.** Quantal Size Estimation and Quantal Parameters at *GluA4*$^{-/-}$ Synapses.

(A) Average data for *N* (*d* = 1.86; p = 8.2E−05), $p_r$ at 2 mM $Ca^{2+}$ (*d* = −2.74; p = 7.6E−05), and EPSC amplitude at 2 mM $Ca^{2+}$ (*d* = −2.1; p = 0.0051) from EPSC amplitude variance-mean analysis for WT and *GluA4*$^{-/-}$. (B) *Left*: Example EPSC amplitude variance-mean relationship illustrating *q* estimation from EPSC amplitude variance at low $Ca^{2+}$ concentration ("limiting slope"). Whereas the parabola fit (black) included all data, the line fit (red) was restricted to the data point with the smallest mean EPSC amplitude for the limiting slope estimate (both fits were constrained to origin). *Right*: Average *q* estimates from limiting slopes of variance-mean analysis for WT and *GluA4*$^{-/-}$ (*d* = −1.88; p = 4.3E−06). (C) Histograms of bootstrapped quantal content for WT and *GluA4*$^{-/-}$ (see *Methods* for details). Quantal content was calculated using EPSC amplitudes recorded at 2 mM $Ca^{2+}$ and *q* from limiting slopes. (D) Bootstrapped *q* estimates from $1/CV^2$ analysis (14) in *GluA4*$^{-/-}$. The *q* estimates from variance-mean analysis (1.22 pA), limiting slope analysis (1.14 pA), and $1/CV^2$ analysis (1.19 pA) agreed well. ** p < 0.01; *** p < 0.001; **** p < 0.0001; n.s. not significant; two-tailed Student's t-test. Boxplots show median and 1st/3rd quartiles, whiskers extend to data within 1.5× the interquartile range from lower or upper quartiles. Bar graphs show mean ± SEM.

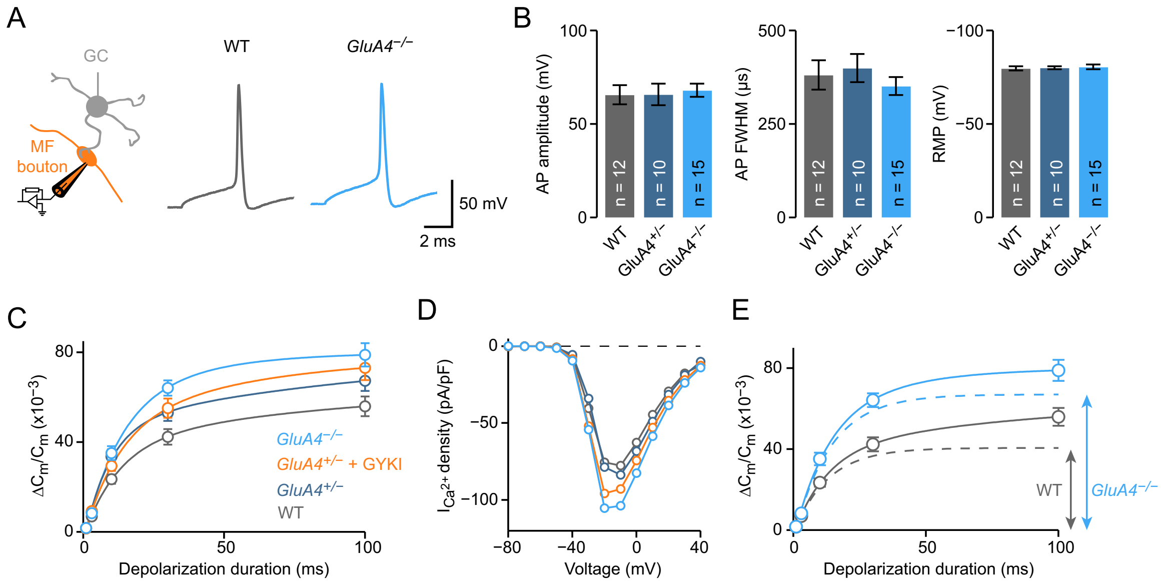


**Fig. S8.** Presynaptic Action Potentials and Exocytosis at *GluA4*-Mutant Synapses.

(A) Representative presynaptic APs elicited by 100-ms depolarizing current injection in cerebellar MF boutons of WT and *GluA4*$^{-/-}$ mice. (B) AP amplitude, AP full width at half maximal amplitude (FWHM), and resting membrane potential (RMP) were similar in boutons of WT, *GluA4*$^{+/-}$, and *GluA4*$^{-/-}$. (C) Membrane capacitance increase ($\Delta C_m$) versus duration of presynaptic depolarization for WT, *GluA4*$^{+/-}$, *GluA4*$^{+/-}$ with GYKI and *GluA4*$^{-/-}$. (D) $Ca^{2+}$-current density for all four conditions; color coding as in C. (E) $\Delta C_m$ versus duration of presynaptic depolarization for WT and *GluA4*$^{-/-}$ (data are replotted from Figure 6). The time course of RRP release could be fitted with a bi-exponential function (WT: $\tau_1$ = 13 ms, $\tau_2$ = 116 ms; *GluA4*$^{-/-}$: $\tau_1$ = 14 ms, $\tau_2$ = 45 ms). Dashed lines indicate the first exponential component for both genotypes. Note the larger amplitude of the fast component in *GluA4*$^{-/-}$ (double arrows), indicating an increase in the number of fast-releasing vesicles (24). ** $p < 0.01$; *** $p < 0.001$; **** $p < 0.0001$; n.s. not significant; two-tailed Student's t-test. Boxplots show median and 1st/3rd quartiles, whiskers extend to data within 1.5× the interquartile range from lower or upper quartiles. Bar graphs represent mean ± SEM.

**Table S1.** Statistics for Main Figures.

| Measurement | n | Effect size * | Test statistic # | df | p value | Test | Figure |
|---|---|---|---|---|---|---|---|
| **Control vs. GYKI** | | | | | | | |
| mEPSC amplitude | 63; 33 | −1.10 | 5.11 | 94 | 1.7E−06 | t test | 1D |
| EPSC amplitude | 63; 33 | −0.02 | 0.08 | 94 | 0.94 | t test | 1D |
| Quantal content | 63; 33 | 0.78 | 3.63 | 94 | 0.00047 | t test | 1D |
| mEPSC amplitude wash | 63; 16 | 0.05 | 0.19 | 77 | 0.85 | t test | 1F |
| EPSC amplitude wash | 63; 16 | 0.74 | 2.63 | 77 | 0.01 | t test | 1F |
| Quantal content wash | 63; 16 | 0.95 | 3.40 | 77 | 0.001 | t test | 1F |
| RRP size | 35; 20 | 0.69 | 2.47 | 53 | 0.017 | t test | 2B |
| $p_r$ train | 35; 20 | −0.04 | 0.14 | 53 | 0.89 | t test | 2B |
| PPR | 16; 18 | 0.05 | 6.55 | 1 | 0.012 | ANOVA | 2D |
| $\Delta C_m$ | 11; 10 | 0.14 | 17.23 | 1 | 6.6E−05 | ANOVA | 2F |
| $Ca^{2+}$ current density | 13; 13 | <0.01 | 0.022 | 1 | 0.88 | ANOVA | 2G |
| **WT vs. *GluA4*$^{+/-}$** | | | | | | | |
| mEPSC amplitude | 31; 50 | −0.90 | 4.05 | 79 | 0.00012 | t test | 3B |
| EPSC amplitude | 31; 43 | 0.14 | 0.60 | 72 | 0.55 | t test | 3B |
| Quantal content | 31; 43 | 0.70 | 2.66 | 72 | 0.0095 | t test | 3B |
| RRP size | 22; 24 | 0.72 | 2.41 | 44 | 0.02 | t test | 3D |
| $p_r$ train | 22; 24 | −0.001 | 0.003 | 44 | 0.99 | t test | 3D |
| $\Delta C_m$ | 21; 19 | 0.07 | 14.56 | 1 | 0.0002 | ANOVA | 3F |
| $Ca^{2+}$ current density | 16; 15 | 0.001 | 0.27 | 1 | 0.60 | ANOVA | 3G |
| ***GluA4*$^{+/-}$ vs. *GluA4*$^{+/-}$ with GYKI** | | | | | | | |
| mEPSC amplitude | 50; 20 | −0.84 | 2.60 | 68 | 0.011 | t test | 4B |
| EPSC amplitude | 43; 20 | −0.14 | 0.46 | 61 | 0.65 | t test | 4B |
| Quantal content | 43; 20 | 0.41 | 1.57 | 61 | 0.12 | t test | 4B |
| RRP size | 24; 16 | −0.11 | 0.35 | 38 | 0.73 | t test | 4C |
| $p_r$ train | 24; 16 | 1.04 | 3.16 | 38 | 0.003 | t test | 4C |
| Tau depression | 24; 16 | −0.90 | 2.79 | 38 | 0.008 | t test | 4D |
| EPSC amplitude CV | 43; 20 | −0.58 | 2.05 | 61 | 0.04 | t test | 4E |
| PPR | 26; 16 | 0.09 | 18.93 | 1 | 2.2E−05 | ANOVA | 4F |
| $\Delta C_m$ | 19; 13 | <0.001 | 0.01 | 1 | 0.92 | ANOVA | 4G |
| $Ca^{2+}$ current density | 16; 15 | 0.02 | 8.22 | 1 | 0.0044 | ANOVA | 4H |
| **WT vs. *GluA4*$^{-/-}$** | | | | | | | |
| EPSC amplitude | 31; 40 | −1.96 | 9.31 | 69 | 8.1E−14 | t test | 5B |
| EPSC tau decay | 31; 40 | 0.84 | 3.12 | 69 | 0.0026 | t test | 5C |
| *q* | 12; 7 | −2.84 | 4.42 | 17 | 0.00037 | t test | 5F |
| Quantal content | 12; 7 | 1.01 | 2.66 | 17 | 0.017 | t test | 5F |
| $\Delta C_m$ | 21; 15 | 0.18 | 39.63 | 1 | 2.2E−09 | ANOVA | 6B |
| $Ca^{2+}$ current density | 15; 12 | 0.07 | 22.51 | 1 | 3.1E−06 | ANOVA | 6D |

* effect size was calculated as Cohen's *d* or partial $\eta^2$ for t test and ANOVA, respectively.
# test statistic represents *T* or *F* for t test and ANOVA, respectively, and is given as absolute value.

**Table S2.** Statistics for Supplementary Figures.

| Measurement | n | Effect size * | Test statistic # | df | p value | Test | Figure |
|---|---|---|---|---|---|---|---|
| **Control vs. GYKI** | | | | | | | |
| mEPSC frequency | 63; 33 | 0.45 | 2.11 | 94 | 0.037 | t test | S1A |
| mEPSC tau decay | 63; 33 | 0.30 | 1.38 | 94 | 0.17 | t test | S1C |
| Single-channel conductance | 12; 14 | −0.17 | 0.45 | 24 | 0.66 | t test | S1E |
| EPSC tau decay | 63; 33 | −0.28 | 1.32 | 94 | 0.19 | t test | S1G |
| EPSC rise time | 63; 33 | −0.41 | 1.94 | 94 | 0.056 | t test | S1G |
| RRP amplitude wash | 35; 13 | 0.83 | 2.56 | 46 | 0.014 | t test | S3B |
| RRP size (EQ) | 35; 20 | 0.86 | 3.08 | 53 | 0.0032 | t test | S3D |
| Recruitment slope | 35; 20 | 0.41 | 1.46 | 53 | 0.15 | t test | S3E |
| EPSC amplitude CV | 63; 33 | −0.39 | 1.80 | 94 | 0.075 | t test | S3F |
| **Control vs. CTZ and KYN** | | | | | | | |
| PPR | 8; 16 | 0.27 | 38.14 | 1 | 1.4E−08 | ANOVA | S3G |
| **mEPSC vs. unitary EPSC** | | | | | | | |
| Amplitude WT | 31; 8 | 0.47 | 1.18 | 37 | 0.25 | t test | S2B |
| Tau decay WT | 31; 8 | 0.17 | 0.43 | 37 | 0.66 | t test | S2B |
| Amplitude *GluA4*$^{+/-}$ | 50; 8 | −0.03 | 0.08 | 56 | 0.94 | t test | S2B |
| Tau decay *GluA4*$^{+/-}$ | 50; 8 | 0.46 | 1.21 | 56 | 0.23 | t test | S2B |
| **WT vs. *GluA4*$^{+/-}$** | | | | | | | |
| mEPSC tau decay | 31; 50 | 0.65 | 2.83 | 79 | 0.0058 | t test | S5B |
| Single-channel conductance | 11; 14 | −0.24 | 0.61 | 24 | 0.55 | t test | S5C |
| mEPSC frequency | 31; 50 | 0.24 | 0.61 | 79 | 0.25 | t test | S5D |
| EPSC tau decay | 31; 43 | 0.38 | 1.55 | 72 | 0.13 | t test | S5E |
| EPSC rise time | 31; 43 | −0.17 | 0.77 | 72 | 0.45 | t test | S5E |
| ***GluA4*$^{+/-}$ vs. *GluA4*$^{+/-}$ with GYKI** | | | | | | | |
| mEPSC tau decay | 50; 20 | −0.97 | 2.94 | 68 | 0.0046 | t test | S5B |
| Single-channel conductance | 14; 14 | 0.21 | 0.51 | 23 | 0.62 | t test | S5C |
| mEPSC frequency | 50; 20 | 0.03 | 0.12 | 68 | 0.91 | t test | S5D |
| EPSC tau decay | 43; 20 | −0.72 | 2.20 | 61 | 0.031 | t test | S5E |
| EPSC rise time | 43; 20 | −0.36 | 1.34 | 61 | 0.19 | t test | S5E |
| **WT vs. *GluA4*$^{+/-}$** | | | | | | | |
| $q$ | 12; 11 | −1.36 | 3.27 | 21 | 0.0037 | t test | S4B |
| $N$ | 12; 11 | 3.23 | 7.74 | 21 | 1.4E−07 | t test | S4B |
| $p_r$ | 12; 10 | −1.77 | 4.13 | 20 | 5.2E−04 | t test | S4B |
| EPSC amplitude | 12; 11 | −0.07 | 0.17 | 21 | 0.87 | t test | S4C |
| Quantal content | 12; 11 | 2.31 | 5.53 | 21 | 1.7E−05 | t test | S4C |
| PPR | 23; 16 | 0.03 | 8.32 | 1 | 0.0043 | ANOVA | S4D |
| EPSC amplitude CV | 31; 43 | −0.19 | 0.81 | 72 | 0.42 | t test | S4E |
| Steady-state EPSC | 22; 24 | 0.15 | 0.51 | 44 | 0.62 | t test | S4F |
| Tau depression | 22; 24 | 0.17 | 0.56 | 44 | 0.58 | t test | S4F |
| Recruitment slope | 22; 24 | 0.29 | 0.99 | 44 | 0.33 | t test | S4F |
| RRP size (EQ) | 22; 24 | 0.72 | 2.38 | 44 | 0.02 | t test | S4G |

| | | | | | | | |
|---|---|---|---|---|---|---|---|
| AP amplitude | 12; 10 | 0.01 | 0.02 | 20 | 0.99 | t test | S7B |
| AP FWHM | 12; 10 | 0.15 | 0.34 | 20 | 0.74 | t test | S7B |
| RMP | 12; 10 | −0.12 | 0.27 | 20 | 0.79 | t test | S7B |
| ***GluA4*$^{+/-}$ vs. *GluA4*$^{+/-}$ with GYKI** | | | | | | | |
| RRP size (EQ) | 24; 16 | −0.90 | 2.79 | 38 | 0.0081 | t test | S5F |
| EPSC amplitude CV vs. pr | 40; 40 | −0.75 | 7.08 | 38 | 1.9E−08 | Pearson r | S5G |
| EPSC amplitude CV vs. RRP | 40; 40 | −0.02 | 0.11 | 38 | 0.91 | Pearson r | S5G |
| **WT vs. *GluA4*$^{-/-}$** | | | | | | | |
| *N* | 12; 4 | 1.86 | 5.14 | 17 | 8.2E−05 | t test | S7A |
| $p_r$ | 12; 7 | −2.74 | 5.17 | 17 | 7.6E−05 | t test | S7A |
| EPSC amplitude | 12; 7 | −2.10 | 3.21 | 17 | 0.0051 | t test | S7A |
| *q* | 11; 16 | −1.88 | 5.84 | 25 | 4.4E−06 | t test | S7B |
| AP amplitude | 12; 15 | 0.15 | 0.39 | 25 | 0.70 | t test | S8B |
| AP FWHM | 12; 15 | −0.26 | 0.67 | 25 | 0.51 | t test | S8B |
| RMP | 12; 15 | −0.18 | 0.47 | 25 | 0.64 | t test | S8B |

* effect size was calculated as Cohen's *d*, partial $\eta^2$ or correlation coefficient for t test, ANOVA, and Pearson, respectively.

# test statistic represents *T* or *F* for t test/correlation and ANOVA, respectively, and is given as absolute value.

**Table S3.** Reagents and Resources.

| Reagent or Resource | Source | Identifier |
|---|---|---|
| **Mouse Strains** | | |
| C57BL/6JRj | Janvier Labs | C57BL/6JRj |
| *GRIA4*$^{-/-}$ ("*GluA4*$^{-/-}$") | Hannah Monyer | (1) |
| **Chemicals** | | |
| 4-AP | Sigma-Aldrich | Cat.# A78403 |
| Bicuculline methiodide | Sigma-Aldrich | Cat.# 14343 |
| Cs-Gluconate | HelloBio | Cat.# HB4822 |
| Cyclothiazide | Tocris | Cat.# 0713 |
| D-APV | Tocris | Cat.# 0106 |
| GYKI 53655 | Tocris | Cat.# 2555 |
| Kynurenic acid | Sigma-Aldrich | Cat.# K3375 |
| Picrotoxin | Sigma-Aldrich | Cat.# P1675 |
| QX-314 chloride | HelloBio | Cat.# HB1030 |
| Strychnine | Sigma-Aldrich | Cat.# S8753 |
| TEA chloride | Sigma-Aldrich | Cat.# T2265 |
| TTX | Tocris | Cat.# 1069 |

## References


1. E. C. Fuchs, *et al.*, Recruitment of Parvalbumin-Positive Interneurons Determines Hippocampal Function and Associated Behavior. *Neuron* **53**, 591–604 (2007).

2. I. Delvendahl, I. Straub, S. Hallermann, Dendritic patch-clamp recordings from cerebellar granule cells demonstrate electrotonic compactness. *Front. Cell. Neurosci.* **9**, 796–8 (2015).

3. A. Ritzau-Jost, *et al.*, Ultrafast action potentials mediate kilohertz signaling at a central synapse. *Neuron* **84**, 152–163 (2014).

4. R. A. Silver, S. G. Cull-Candy, T. Takahashi, Non-NMDA glutamate receptor occupancy and open probability at a rat cerebellar synapse with single and multiple release sites. *J. Physiol.* **494**, 231–250 (1996).

5. S. Hallermann, *et al.*, Bassoon Speeds Vesicle Reloading at a Central Excitatory Synapse. *Neuron* **68**, 710–723 (2010).

6. R. A. Silver, S. F. Traynelis, S. G. Cull-Candy, Rapid-time-course miniature and evoked excitatory currents at cerebellar synapses in situ. *Nature* **355**, 163–166 (1992).

7. R. Schneggenburger, A. C. Meyer, E. Neher, Released fraction and total size of a pool of immediately available transmitter quanta at a calyx synapse. *Neuron* **23**, 399–409 (1999).

8. L. Cathala, N. B. Holderith, Z. Nusser, D. A. DiGregorio, S. G. Cull-Candy, Changes in synaptic structure underlie the developmental speeding of AMPA receptor-mediated EPSCs. *Nat. Neurosci.* **8**, 1310–1318 (2005).

9. J. S. Rothman, R. A. Silver, NeuroMatic: An Integrated Open-Source Software Toolkit for Acquisition, Analysis and Simulation of Electrophysiological Data. *Front. Neuroinformatics* **12**, 1159–21 (2018).

10. P. B. Sargent, Rapid Vesicular Release, Quantal Variability, and Spillover Contribute to the Precision and Reliability of Transmission at a Glomerular Synapse. *J. Neurosci.* **25**, 8173–8187 (2005).

11. C. Saviane, R. A. Silver, “Estimation of Quantal Parameters With Multiple-Probability Fluctuation Analysis” in *Patch-Clamp Methods and Protocols*, Methods in Molecular Biology™, P. Molnar, J. J. Hickman, Eds. (Humana Press, 2007), pp. 303–317.

12. J. D. Clements, R. A. Silver, Unveiling synaptic plasticity: a new graphical and analytical approach. *Trends Neurosci.* **23**, 105–113 (2000).

13. B. Efron, R. Tibshirani, Bootstrap methods for standard errors, confidence intervals, and other measures of statistical accuracy. *Stat. Sci.* **1**, 54–77 (1986).

14. Y. Sahara, T. Takahashi, Quantal components of the excitatory postsynaptic currents at a rat central auditory synapse. *J. Physiol.* **536**, 189–197 (2001).

15. S. F. Traynelis, R. A. Silver, S. G. Cull-Candy, Estimated conductance of glutamate receptor channels activated during EPSCs at the cerebellar mossy fiber-granule cell synapse. *Neuron* **11**, 279–289 (1993).

16. I. Delvendahl, N. P. Vyleta, H. von Gersdorff, S. Hallermann, Fast, Temperature-Sensitive and Clathrin-Independent Endocytosis at Central Synapses. *Neuron* **90**, 492–498 (2016).

17. I. Delvendahl, *et al.*, Reduced endogenous $Ca^{2+}$ buffering speeds active zone $Ca^{2+}$ signaling. *Proc. Natl. Acad. Sci.* **112**, E3075–E3084 (2015).

18. M. Lindau, E. Neher, Patch-clamp techniques for time-resolved capacitance measurements in single cells. *Pflüg. Arch. Eur. J. Physiol.* **411**, 137–146 (1988).

19. S. Hallermann, C. Pawlu, P. Jonas, M. Heckmann, A large pool of releasable vesicles in a cortical glutamatergic synapse. *Proc. Natl. Acad. Sci.* **100**, 8975–8980 (2003).

20. R. Heidelberger, Multiple Components of Membrane Retrieval in Synaptic Terminals Revealed by Changes in Hydrostatic Pressure. *J. Neurophysiol.* **88**, 2509–2517 (2002).

21. R Development Core Team, *R: A Language and Environment for Statistical Computing* (R Foundation for Statistical Computing, 2008).

22. D. Elmqvist, D. M. Quastel, A quantitative study of end-plate potentials in isolated human muscle. *J. Physiol.* **178**, 505–529 (1965).

23. C. Saviane, R. A. Silver, Fast vesicle reloading and a large pool sustain high bandwidth transmission at a central synapse. *Nature* **439**, 983–987 (2006).

24. T. Sakaba, E. Neher, Calmodulin mediates rapid recruitment of fast-releasing synaptic vesicles at a calyx-type synapse. *Neuron* **32**, 1119–1131 (2001).
